# Lung penetration, bronchopulmonary pharmacokinetic/pharmacodynamic profile and safety of 3 g of ceftolozane/tazobactam administered to ventilated, critically ill patients with pneumonia

**DOI:** 10.1093/jac/dkaa049

**Published:** 2020-03-24

**Authors:** Luzelena Caro, David P Nicolau, Jan J De Waele, Joseph L Kuti, Kajal B Larson, Elaine Gadzicki, Brian Yu, Zhen Zeng, Adedayo Adedoyin, Elizabeth G Rhee

**Affiliations:** d1 Merck & Co., Inc., Kenilworth, NJ, USA; d2 Center for Anti-Infective Research and Development, Hartford Hospital, Hartford, CT, USA; d3 Department of Critical Care Medicine, Ghent University Hospital, Ghent, Belgium

## Abstract

**Objectives:**

Ceftolozane/tazobactam is approved for hospital-acquired/ventilator-associated bacterial pneumonia at double the dose (i.e. 2 g/1 g) recommended for other indications. We evaluated the bronchopulmonary pharmacokinetic/pharmacodynamic profile of this 3 g ceftolozane/tazobactam regimen in ventilated pneumonia patients.

**Methods:**

This was an open-label, multicentre, Phase 1 trial (clinicaltrials.gov: NCT02387372). Mechanically ventilated patients with proven/suspected pneumonia received four to six doses of 3 g of ceftolozane/tazobactam (adjusted for renal function) q8h. Serial plasma samples were collected after the first and last doses. One bronchoalveolar lavage sample per patient was collected at 1, 2, 4, 6 or 8 h after the last dose and epithelial lining fluid (ELF) drug concentrations were determined. Pharmacokinetic parameters were estimated by non-compartmental analysis and pharmacodynamic analyses were conducted to graphically evaluate achievement of target exposures (plasma and ELF ceftolozane concentrations >4 mg/L and tazobactam concentrations >1 mg/L; target in plasma: ≥30% and ≥20% of the dosing interval, respectively).

**Results:**

Twenty-six patients received four to six doses of study drug; 22 were included in the ELF analyses. Ceftolozane and tazobactam *T*_max_ (6 and 2 h, respectively) were delayed in ELF compared with plasma (1 h). Lung penetration, expressed as the ratio of mean drug exposure (AUC) in ELF to plasma, was 50% (ceftolozane) and 62% (tazobactam). Mean ceftolozane and tazobactam ELF concentrations remained >4 mg/L and >1 mg/L, respectively, for 100% of the dosing interval. There were no deaths or adverse event-related study discontinuations.

**Conclusions:**

In ventilated pneumonia patients, 3 g of ceftolozane/tazobactam q8h yielded ELF exposures considered adequate to cover ceftolozane/tazobactam-susceptible respiratory pathogens.

## Introduction

Critically ill patients with nosocomial pneumonia are challenging to treat: they exhibit notable alterations in antimicrobial pharmacokinetics (PK) and often have multiorgan dysfunction, immune system dysfunction and/or infections caused by resistant pathogens; these factors all contribute to poor clinical outcomes.^1–4^ Nosocomial pneumonia, in particular ventilator-associated bacterial pneumonia (VABP) and ventilated hospital-acquired bacterial pneumonia (vHABP), is associated with high mortality, which reaches >40% in VABP caused by *Pseudomonas aeruginosa* and in vHABP.^5–7^ Since patients with pneumonia who require mechanical ventilation are at high risk of mortality and other adverse outcomes, they must promptly receive appropriate antibacterial therapy.^8–13^*P. aeruginosa*, including MDR strains, and Enterobacteriaceae (new taxonomy: Enterobacterales), including ESBL-producing strains, are the most common Gram-negative pathogens implicated in this setting.^14^ Given the prevalence of drug-resistant Gram-negative pathogens in nosocomial pneumonia, new safe and broadly effective treatment options that can reach sufficient concentrations at the target site are urgently needed.^4^

Ceftolozane/tazobactam is a cephalosporin/β-lactamase inhibitor combination consisting of ceftolozane, a potent antipseudomonal (including most MDR strains) cephalosporin, and tazobactam, an established β-lactamase inhibitor expanding ceftolozane’s activity against Enterobacteriaceae producing certain ESBLs.^15–19^ Both ceftolozane and tazobactam have dose-proportional, linear, independent PK. Both are renally eliminated, ceftolozane almost exclusively as unchanged drug and tazobactam partially (20%) as the pharmacologically inactive metabolite tazobactam M1; with normal renal function or mild renal impairment, elimination half-lives in patients (derived from population PK modelling) are approximately 3–4 h and 2–3 h, respectively.^20^ The lack of drug–drug interaction between both agents probably occurs because ceftolozane is almost exclusively eliminated by passive glomerular filtration, so it does not interfere with the active tubular secretion of tazobactam.^16^^,^^20^^,^^21^ For treatment of ventilated nosocomial pneumonia, ceftolozane/tazobactam is approved at a dose of 3 g (2 g of ceftolozane and 1 g of tazobactam) q8h, adjusted based on renal function, which is double the dose recommended for other indications.^20^ This higher dose was projected to achieve 98% PTA in pulmonary epithelial lining fluid (ELF) against pathogens with a ceftolozane/tazobactam MIC of ≤8 mg/L,^22^ based on prospective data showing good intrapulmonary penetration in healthy volunteers.^23^ In the recently completed ASPECT-NP Phase 3 trial, the 3 g ceftolozane/tazobactam regimen was non-inferior to meropenem for treatment of HABP/VABP in both primary and key secondary endpoints.^24^

Since bronchopulmonary disposition in critically ill patients could be different compared with healthy volunteers,^4^^,^^25–27^ it is important to evaluate the exposure profile of ceftolozane/tazobactam specifically in this high-risk population.^28^^,^^29^ To further evaluate the clinical utility of the 3 g dose of ceftolozane/tazobactam in nosocomial pneumonia, we conducted a clinical trial to assess the bronchopulmonary penetration of both ceftolozane and tazobactam in mechanically ventilated patients with pneumonia.

## Patients and methods

### Study design

Protocol MK-7625A-007 was a prospective, open-label, multicentre, Phase 1 study characterizing PK, safety and tolerability of ceftolozane/tazobactam in critically ill adults, with two patient cohorts (clinicaltrials.gov: NCT02387372). Herein, we report results from the first cohort, which comprised mechanically ventilated patients concurrently receiving standard-of-care antibacterial therapy for proven or suspected pneumonia. Patients were enrolled at 10 study sites [mostly medical or surgical ICUs; Table S1 (available as Supplementary data at *JAC* Online)] in Belgium, Spain and the USA between May 2015 and June 2017. The full study protocol is also available as Supplementary data at *JAC* Online.

### Ethics

The study was conducted in accordance with principles of Good Clinical Practice and was approved by the appropriate regulatory agencies. Ethics approval was obtained from each participating institution’s appropriate institutional review board (see Table S1 for the specific independent ethics committees). Each patient, or a legal representative, provided written informed consent and was able to withdraw from the study at any time.

### Patients

Patients were ≥18 years old with proven/suspected pneumonia who were hospitalized in the ICU while receiving mechanical ventilation for ≥24 h before enrolment and were expected to continue ventilation for ≥8 h following the final study dose. Proven/suspected pneumonia was defined as the presence of at least one of the following: fever (body temperature >38.5°C), hypothermia (body temperature <35.0°C), elevated WBC count ≥12 000 cells/mm^3^ or chest radiograph showing pneumonia characteristics. Patients had to be receiving antibacterial therapy for pneumonia at enrolment and likely to require continued antibacterial therapy while in the study. Patients were excluded for: baseline haemoglobin levels <7 g/dL; end-stage renal disease, defined as CL_CR_ <15 mL/min (calculated using the Cockcroft–Gault equation based on actual body weight) and/or requirement for continuous renal replacement therapy or haemodialysis; prior (≤24 h before first study dose) or concomitant receipt of piperacillin/tazobactam, non-study ceftolozane/tazobactam or probenecid; or conditions that may interfere with PK assessment or interpretation (e.g. cystic fibrosis, acute exacerbation of chronic bronchitis or obstructive airway disease, chronic severe respiratory disease, active pulmonary TB or lung transplant).

### Study drug

Eligible patients received four to six doses of ceftolozane/tazobactam q8h, administered as 60 min IV infusions. Dosage was adjusted for renal function: 3 g of ceftolozane/tazobactam (2 g of ceftolozane and 1 g of tazobactam) for patients with CL_CR_ >50 mL/min; 1.5 g (1 g of ceftolozane and 500 mg of tazobactam) for patients with CL_CR_ between 30 and 50 mL/min; and 750 mg (500 mg of ceftolozane and 250 mg tazobactam) for patients with a CL_CR_ between 15 and 29 mL/min. CL_CR_ was calculated once daily, at a consistent time, while receiving study drug. Patients receiving the lowest ceftolozane/tazobactam regimen needed to receive six doses to achieve steady-state plasma concentrations, while for all other patients the number of doses was at the investigator’s discretion to facilitate timing of bronchoalveolar lavage (BAL).

### Plasma and ELF sample collection

Blood samples for plasma concentration determination of ceftolozane and tazobactam were collected at 0 (pre-dose), 1, 2, 4, 6 and 8 h after start of infusion during the first and final (i.e. fourth, fifth or sixth) doses of ceftolozane/tazobactam. At least 5.0 mL of blood was collected per timepoint using a sodium heparin vacutainer tube, which was then mixed by inverting five times and kept on ice after collection. Blood samples were centrifuged at approximately 4°C within 30 min of collection at 3000 rpm (approximately 2056 **g** force) for 15–20 min to achieve a clear plasma layer over the red cells. Plasma samples were subsequently aliquotted into pre-chilled polypropylene cryotubes and immediately frozen and stored at −80 ± 10°C in an upright position.

All patients were randomly assigned to undergo one bronchoscopy and BAL (of the lung segment identified as containing the pneumonia) at 1 (±30 min), 2 (±30 min), 4 (±30 min), 6 (±30 min) or 8 (±30 min) h after start of the final study drug infusion (*n = *5 per timepoint). Each BAL utilized four aliquots of sterile 0.9% saline: the initial aliquot (50 mL) was discarded and the subsequent aliquots (20 mL each) were stored on ice, pooled and centrifuged for 5 min at 400 **g** force at approximately 4°C. Immediately after centrifugation, the supernatant was aliquotted into three 15 mL amber-coloured specimen bottles, which were frozen at −80 ± 10°C within 17 h of sample collection and then shipped to a central laboratory.

Safety and tolerability were assessed throughout the study by examining adverse events (AEs), changes in clinical laboratory test results and changes in vital signs. AEs were collected throughout the study until 24–48 h after the last dose of study drug.

### Drug concentration determination

Concentrations of ceftolozane and tazobactam in plasma and BAL fluid were determined at MicroConstants (San Diego, CA, USA), using a validated HPLC–tandem MS method described in detail previously.^23^^,^^30^ For plasma, the analytical ranges were 0.250 to 150 mg/L (ceftolozane) and 0.100 to 60.0 mg/L (tazobactam). For BAL fluid, the analytical ranges were 1.00 to 1000 ng/mL (ceftolozane) and 1.00 to 250 ng/mL (tazobactam), with accuracy of ∼95.5% (ceftolozane) and ∼94.5% (tazobactam) and precision ranging from 6.27% to 7.78% and 5.37% to 10.1%, respectively. To account for dilution from the saline instillations during BAL, drug concentrations in ELF were estimated by correcting the respective BAL concentrations using the urea dilution method.^31^ Urea concentrations were also determined at MicroConstants, using an unmodified version of the colorimetric BioChain Urea Assay Kit (BioChain Institute Inc., Newark, CA, USA). This validated urea assay had an analytical range of 2.50 to 50.0 mg/dL (plasma) and 0.150 to 2.50 mg/dL (BAL fluid) and for BAL fluid had an accuracy ranging from 95.00% to 98.50% and precision ranging from 7.54% to 10.9%. For BAL samples without detectable urea concentrations using this assay, a high-sensitivity assay with an analytical range of 0.0500 to 1.00 mg/dL was subsequently used; the high-sensitivity assay was also based on the BioChain Urea Assay Kit, but used 300 μL of sample and 400 μL of chromogenic solution (instead of 75 μL and 200 μL, respectively, for the unmodified assay). Urea concentrations were assessed from the same samples as drug concentrations.

### PK and pharmacodynamic (PD) analyses

PK parameters were estimated by non-compartmental analyses (NCAs) using Phoenix WinNonlin version 6.3 (Certara, Princeton, NJ, USA). The AUC was calculated using the linear up/log down method. Plasma PK parameters were calculated using actual sampling times. Individual ELF concentrations measured at each timepoint (i.e. 1, 2, 4, 6 and 8 h post-dose) were averaged (as geometric means) across all patients with data from that particular timepoint; these pooled, mean concentrations were used to compile one ELF concentration–time profile from which the ELF PK parameter values were estimated using NCA. When conducting this NCA, average 0 h pre-dose concentrations were assumed to be the same as average 8 h post-dose concentrations. Since in ELF only a single PK profile was created for ceftolozane and tazobactam, statistical measures of variability could not be calculated. All plasma drug concentrations are reported as unbound plasma concentrations, estimated assuming 21% protein binding for ceftolozane and 30% for tazobactam.^20^ In ELF, 0% protein binding was assumed for both ceftolozane and tazobactam, and all ELF drug concentration are reported as total ELF concentrations. Since the dose was adjusted based on CL_CR_, drug exposure was expected to be similar across dosing regimens and individual ceftolozane and tazobactam PK results were pooled across all dose levels for analysis. Ceftolozane and tazobactam lung penetration was calculated as the ratio of the mean AUC_0–8_ in ELF to the mean unbound AUC_0–8_ in plasma.

We also evaluated graphically how individual plasma concentration–time profiles (for both first and last doses) and the mean ELF concentration–time profiles (for last dose) related to specific target concentrations. The established PK/PD target for ceftolozane, based on previously published work, is the free plasma ceftolozane concentration remaining at >4 mg/L (the CLSI susceptibility breakpoint for *P. aeruginosa*) for ≥30% of the 8 h dosing interval; as a sensitivity analysis we also assessed a PK/PD target of >8 mg/L (the CLSI intermediate susceptibility breakpoint for *P. aeruginosa*).^32^^,^^33^ The established PK/PD target for tazobactam is the free tazobactam plasma concentration remaining at >1 mg/L (threshold concentration needed to effectively neutralize susceptible bacterial β-lactamases) for ≥20% of the dosing interval.^34^ For ELF, the duration that absolute ceftolozane and tazobactam exposures remained above these respective concentration thresholds was explored.

## Results

### Patients

Of the 27 patients enrolled, 26 (96%) received ceftolozane/tazobactam; 1 patient was extubated prior to treatment assignment and was discontinued from the study without receiving study drug. In these 26 patients, CL_CR_ ranged from 37.7 to 355.6 mL/min, including 8 patients (30.8%) with augmented renal clearance (defined as CL_CR_ values >150 mL/min). Twenty-one patients received 3 g of ceftolozane/tazobactam, four received 1.5 g of ceftolozane/tazobactam (dosing of one patient was adjusted from 1.5 to 3 g for the fourth and fifth doses because of increased CL_CR_ post-baseline) and one received 750 mg of ceftolozane/tazobactam.

Among the 26 patients who received ceftolozane/tazobactam, 25 provided data for plasma PK analyses and 22 provided data for BAL PK analyses. One patient was excluded from the plasma PK analyses due to having received an incorrectly adjusted ceftolozane/tazobactam dose (i.e. 1.5 g of ceftolozane/tazobactam despite having had a CL_CR_ >50 mL/min). This patient was also excluded from the BAL PK analyses, along with three other patients who either had no BAL performed (one was extubated prior to the last dose and another was deemed too clinically unstable to undergo the procedure) or had no urea detected in BAL fluid (one patient). Baseline demographic and clinical characteristics of the 22 patients comprising the ELF PK population are summarized in Table 1.

**Table 1.** Baseline demographics and clinical characteristics of patients included in the ELF PK analysis (*N = *22)

Baseline demographics	
age (years), mean±SD (range)	63.0±16.3 (21–88)
male, *n* (%)	15 (68)
race, *n* (%)	
white	16 (73)
black	6 (27)
weight (kg), mean±SD (range)	89.6±25.3 (46.0–142.0)
BMI (kg/m^2^), mean±SD (range)	30.6±9.3 (13.8–55.5)
CL_CR_ (mL/min), mean±SD (range)	121.5±76.6 (23.7–355.6)
Reason for ICU admission, *n* (%)	
cardiac disorders^a^	5 (23)
gastrointestinal disorders^b^	1 (5)
injuries^c^	4 (18)
infections^d^	2 (9)
nervous system disorders^e^	3 (14)
psychiatric disorders^f^	1 (5)
respiratory, thoracic and mediastinal disorders^g^	6 (27)
Pneumonia diagnosis, *n* (%)	
confirmed	20 (91)
suspected	2 (9)

a Cardiac arrest (*n = *4) and cardiopulmonary arrest.

b Gastrointestinal haemorrhage.

c Road traffic accident, gunshot wound, traumatic brain injury and subdural haematoma.

d Severe sepsis (secondary to community-acquired pneumonia) and sepsis (secondary to empyema).

e Cerebral artery stroke, intraparenchymal haemorrhage and neurological deterioration.

f Altered mental status.

g Respiratory failure (*n = *2), aspiration pneumonia, dyspnoea, haemothorax and massive haemoptysis.

### PK and PD analyses

**Figure 1.**
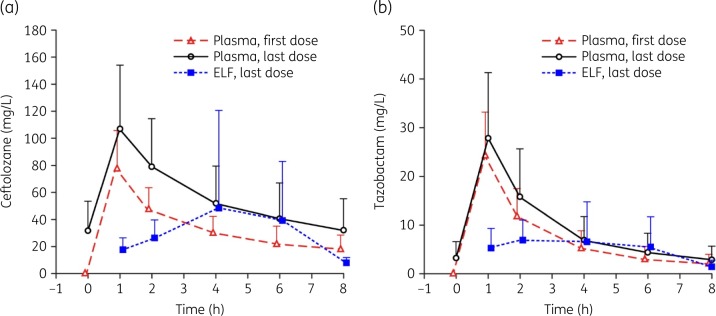
Arithmetic mean (±SD) total concentration–time profiles in plasma [first dose (*n = *25 patients) and last dose (*n = *24 patients)] and ELF (last dose; *n = *22 patients) for (a) ceftolozane and (b) tazobactam. Note: curves are slightly offset around each timepoint in order to achieve separation of error bars.

Plasma and ELF PK parameter estimates are presented in Table 2 as geometric means. Plasma accumulation from the first to the last dose was ∼1.6- and 1.2-fold for ceftolozane and tazobactam AUC_0–8_, respectively. The median ceftolozane and tazobactam *T*_max_ in plasma were 1 h for both the first and last doses (Table 2 and Figure 1). Ceftolozane and tazobactam *T*_max_ in ELF after the last dose were 6 and 2 h, respectively (Table 2 and Figure 1). Lung penetration was approximately 50% for ceftolozane and 62% for tazobactam.

**Table 2.** PK parameters for ceftolozane and tazobactam, presented as geometric mean (geometric coefficient of variation %) unless otherwise noted, in plasma and ELF among ventilated, critically ill patients with pneumonia who received 3 g of ceftolozane/tazobactam (dose-adjusted for renal impairment) q8h for four to six doses

PK parameter	Plasma		ELF^d^, last dose (*N *=* *22)
	first dose (*N *=* *25)	last dose (*N *=* *24)^a^	
Ceftolozane			
AUC_0–8_ (mg·h/L)	248 (40.6)^b^	392 (57.8)^c^	154
*C*_max_ (mg/L)	73.0 (42.3)	101 (45.9)	27.4
*T*_max_ (h), median (range)	1.00 (0.88–2.00)	1.00 (0.92–2.17)	6^e^
*C*_last_ (mg/L)	14.0 (70.7)	23.8 (100.0)	7.54
*V*_ss_ (L)	27.5 (29.9)^b^	29.4 (45.5)^c^	NA
CL (L/h)	4.90 (64.1)^b^	4.52 (67.7)^c^	NA
*t*_½_ (h)	4.15 (56.1)^b^	4.86 (61.5)^c^	NA
Tazobactam			
AUC_0–8_ (mg·h/L)	51.3 (49.4)	63.0 (64.8)^c^	27.5
*C*_max_ (mg/L)	22.6 (41.1)	26.2 (50.1)^c^	5.37
*T*_max_ (h), median (range)	1.00 (0.88–1.12)	1.00 (0.92–2.17)	2^e^
*C*_last_ (mg/L)	1.14 (147.4)	1.49 (169.9)	1.11
*V*_ss_ (L)	39.9 (28.9)	40.4 (38.8)^c^	NA
CL (L/h)	15.0 (68.5)	14.1 (75.6)^c^	NA
*t*_½_ (h)	2.15 (56.4)	2.33 (49.7)^c^	NA

AUC_0–8_, area under the concentration–time curve from time 0 to 8 h (i.e. the dosing interval); *C*_max_, maximum observed concentration; *T*_max_, time to maximum observed concentration; *C*_last_, last quantifiable concentration; *V*_ss_, volume of distribution at steady-state; CL, clearance; *t*_½_, terminal half-life; NA, not available.

a One patient was extubated prior to the final plasma PK sampling.

b Data available from 24 patients: 1 patient had the last timepoint collected after the next dose, so PK data for the last timepoint were excluded.

c Data available from 23 patients: 1 patient received a commercial product instead of the study drug at 6 h, so PK data for the last timepoint were excluded.

d ELF data were pooled at each timepoint to allow PK parameters to be generated. Since a single, pooled PK profile was determined in ELF, statistical measures of variability cannot be calculated for any ceftolozane and tazobactam PK parameters in ELF.

e This *T*_max_ corresponds to the time when the pooled ELF concentration-time profile, calculated based on geometric mean values at each timepoint, reached the maximum concentration.

**Figure 2.**
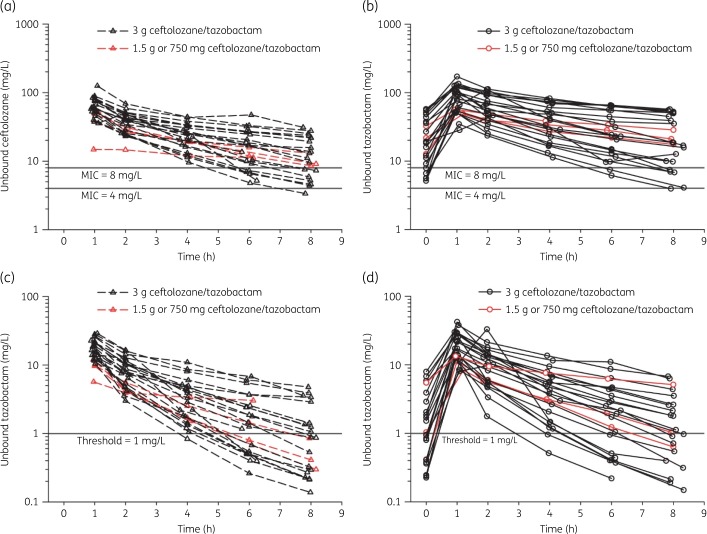
Individual unbound plasma concentration–time profiles for first (*n = *25) and last (*n = *24) doses, with target concentrations shown as horizontal lines, among patients included in the plasma PK analyses, for (a) ceftolozane first dose, (b) ceftolozane last dose, (c) tazobactam first dose and (d) tazobactam last dose.

After the first and last doses, all patients had drug exposures in plasma that were above the PK/PD targets for both ceftolozane and tazobactam. Figure 2 illustrates that plasma exposures above these targets were rapidly achieved for both ceftolozane and tazobactam in all patients and sustained for the duration of the entire dosing interval in most patients. Following the last dose, the geometric mean unbound plasma ceftolozane concentration remained >4 mg/L for approximately 100% (range: 98%–100%) and >8 mg/L for approximately 96% (range: 64%–100%) of the dosing interval and the geometric mean unbound tazobactam plasma concentration remained >1 mg/L for approximately 82% (range: 35%–100%) of the dosing interval (Table S2). In patients with augmented renal clearance, geometric mean total ceftolozane concentrations in plasma remained >4 mg/L for 99.7% and mean total tazobactam concentrations >1 mg/L for 75.5% of the dosing interval following the last dose. In ELF, arithmetic and geometric mean total ceftolozane and tazobactam concentrations remained >4 mg/L (and also >8 mg/L in a sensitivity analysis) and >1 mg/L, respectively, for 100% of the dosing interval following the last dose (Figure S1 and Table S2); this was also the case for patients with augmented renal clearance.

Figure S2 shows individual drug concentrations for patients in the ELF PK population who received renally adjusted doses of ceftolozane/tazobactam (i.e. <3 g q8h) compared with average concentrations in patients with normal renal function (who received 3 g q8h). Individual ceftolozane and tazobactam concentrations in these renally impaired patients generally fell within the 5th and 95th percentiles of the median concentrations observed in patients with normal renal function, suggesting that the dose adjustments made according to renal function were adequate to allow pooling of the data. The appropriateness of pooling these data was also confirmed by the individual plasma concentration–time profiles, which were similar between both groups of patients.

### Safety

There were 43 treatment-emergent AEs experienced by 16 of the 26 patients (61.5%) who received ceftolozane/tazobactam; all of these AEs were mild to moderate in severity (Table S3). There were no deaths or serious AEs. No patients discontinued the study due to an AE. The most frequent treatment-emergent AEs, and the only ones reported in at least two patients, were anaemia and diarrhoea (Table S4). Two patients had treatment-related AEs: moderate thrombocytopenia (*n = *1) and moderate diarrhoea (*n = *1), both of which resolved by the final study visit.

## Discussion

In patients with nosocomial pneumonia, drug exposure in the lungs is recognized as a crucial factor for determining dosing regimens likely to result in good clinical outcomes.^28^ Ceftolozane/tazobactam is a cephalosporin/β-lactamase inhibitor combination recently approved for treating nosocomial pneumonia using a 3 g q8h regimen, which is double the dose recommended for other indications.^20^ Since critically ill patients often have differences in drug PK and tissue distribution compared with healthy individuals,^26–29^^,^^35^ it is important to evaluate bronchopulmonary concentrations specifically in this group of patients.^28^^,^^36^ The purpose of our unique and rigorously conducted clinical trial was to assess whether the 3 g dose would provide adequate lung penetration to achieve ceftolozane and tazobactam concentrations above relevant target thresholds, for which an NCA was sufficient. The data from this study will be combined with a larger set of sparse PK data from the recently completed large, randomized, controlled, ASPECT-NP^24^ trial using a population PK approach and subsequent modelling and target attainment simulations.^36^ Of note, in ASPECT-NP, which was conducted exclusively in patients with ventilated nosocomial pneumonia, the 3 g dose of ceftolozane/tazobactam was shown to be efficacious for the treatment of HABP/VABP.

Our results demonstrated that lung penetration for both ceftolozane and tazobactam (50% and 62%, respectively, based on mean PK profiles) was similar between the critically ill, mechanically ventilated patients with pneumonia enrolled in our trial and the healthy volunteers previously studied (61% and 63%, respectively, assuming 21% and 30% plasma protein binding, respectively).^23^ Of note, steady-state plasma AUC, *C*_max_ and terminal half-life for both ceftolozane and tazobactam observed in our study population of ventilated, critically ill pneumonia patients were very similar to the respective population PK-derived values from the ASPECT-NP clinical trial in mechanically ventilated patients with HABP/VABP and CL_CR_ >50 mL/min.^20^ Importantly, mean ceftolozane and tazobactam ELF concentrations in this pharmacologically complex, high-risk population remained above 8 mg/L and 1 mg/L, respectively, for 100% of the dosing interval. Our results also demonstrated that the 3 g q8h ceftolozane/tazobactam regimen achieved established PK/PD targets [i.e. ceftolozane plasma concentrations remaining above 4 mg/L (and also above 8 mg/L) for at least 30% and tazobactam concentrations remaining above 1 mg/L for at least 20% of the dosing interval].^32^^,^^34^ These thresholds were also met in patients with augmented renal clearance. (Previously published population PK analyses^37^^,^^38^ and a clinical trial in HABP/VABP^24^ also support the notion that the presence of augmented renal clearance does not necessitate ceftolozane/tazobactam dose adjustments.) Furthermore, the 3 g dose was generally well tolerated, just as in other clinical trials assessing this higher regimen,^21^^,^^24^^,^^30^^,^^39^ with no AEs reported as serious, severe or resulting in treatment discontinuation.

Interpatient variability in plasma PK was higher in these critically ill patients, which is not surprising given the generally more complex physiological variation in this population.^26–29^ Drug distribution, as a function of time to reach maximum drug concentration in ELF, was delayed in critically ill pneumonia patients versus healthy volunteers,^23^ but overall ELF drug exposures per dosing interval were similar in both study populations. These results suggest that certain pathophysiological factors (e.g. capillary leakage or fluid loading) in patients with pneumonia may delay ceftolozane/tazobactam distribution into ELF; however, the overall effect on ceftolozane/tazobactam lung penetration and, most important, on the critical PK/PD parameters of time above ceftolozane and tazobactam target concentrations, appears not to be clinically relevant. Taken as a whole, these bronchopulmonary disposition data suggest that the 3 g dose regimen provides adequate drug exposure in ventilated, critically ill patients with pneumonia. Of note, volume of distribution is often much greater in critically ill patients with infections than in healthy volunteers and non-critically ill patients.^27^ This was the case with our study participants (plasma *V*_ss_ ≈29 L) compared with healthy volunteers who also received multiple doses of 3 g of ceftolozane/tazobactam q8h (plasma *V*_ss_ ≈18 L).^30^

Although ELF concentration data have previously been reported for various antibacterial agents, including other novel β-lactam/β-lactamase inhibitor combinations, most such assessments of antibacterial lung penetration were only conducted in healthy volunteers.^23^^,^^40–43^ In our study, the rigour of intensive plasma PK sampling and structured timing of plasma and BAL sample collection were similar to methods employed in healthy volunteer studies, but were applied to a study population intended to be representative of real-world patients encountered in clinical practice (including those with augmented renal clearance or renal dysfunction) and similar to the study population of a Phase 3 trial that evaluated ceftolozane/tazobactam for nosocomial pneumonia. Therefore, patients with a range of renal function were eligible, as were patients receiving concomitant medications not expected to interfere with the PK of ceftolozane or tazobactam. Furthermore, patients were enrolled worldwide at multiple sites and a standardized BAL sampling procedure was outlined to minimize intersite sampling variability.

Nonetheless, the high variability we observed in the plasma and ELF PK data (particularly high for plasma half-life) was anticipated, due to the physiological complexity of our study population compared with healthy volunteers. Since ceftolozane/tazobactam is mainly renally eliminated, and CL_CR_ is therefore the primary variable influencing and predicting exposure, this observed plasma variability was probably due to differences in patients’ baseline renal function (as evident by the wide range of baseline CL_CR_ levels). A comparison of drug concentrations in patients with normal versus those with impaired renal function showed that it was appropriate to pool all patients regardless of whether or not they underwent CL_CR_-based dose adjustments, based on the similarities between the respective ceftolozane and tazobactam exposures. This approach is supported by previous studies that demonstrated similar drug exposures across the approved ceftolozane/tazobactam dose categories.^38^^,^^44^^,^^45^ CL_CR_, calculated once daily, was a suitable marker for renal function because it reflects exactly how ceftolozane/tazobactam should be used in clinical practice.^20^ (Of note, there is evidence that renal function-based dose adjustments made during the first 48–72 h of antibacterial treatment could result in underdosing, because more than half of all patients with renal impairment at treatment initiation appear to improve renal function within that time frame.^46^ This further highlights the importance of daily CL_CR_ estimation during ceftolozane/tazobactam treatment^20^—paying close attention to likely renal function improvements in patients with acute kidney injury and CL_CR_-based dose adjustments will allow for rapid escalation to the standard dosing regimen once appropriate.) Additionally, all but one patient had stable renal function from baseline throughout the study. High interpatient variability was also noted in ELF concentrations at various timepoints, with a concentration difference greater than ∼10-fold at some timepoints (e.g. at 4 h post-dose). Given that only one BAL sample for evaluation of ceftolozane/tazobactam ELF concentrations could be collected per patient, a composite ELF concentration–time profile was used. Current study designs for ELF sampling have limitations and can lead to overestimation of variability in ELF concentration data.^36^ It was therefore not possible to determine whether the high interpatient variability observed at specific timepoints was reflective of high interpatient variability in PK parameters (e.g. AUC or half-life) or patient characteristics (e.g. disease state, age or renal function). The ELF variability we observed in critically ill patients appeared to be greater than that previously reported from healthy volunteers.^23^ Of note, results from a porcine pneumonia model suggest that a single BAL sampling timepoint is sufficient to predict the median penetration and variability for ceftolozane.^47^ Therefore, although significant interpatient variability was observed in our study, it is reasonable to use the mean profile from pooled ELF data to assess drug exposure. In addition, all ceftolozane and tazobactam ELF concentrations observed during the dosing interval remained above target concentrations, further supporting use of the pooled profile.

In conclusion, in these ventilated, critically ill patients with pneumonia, 3 g of ceftolozane/tazobactam was generally well tolerated and yielded ELF exposures sufficient to treat susceptible Gram-negative lower respiratory tract pathogens with ceftolozane/tazobactam MIC values up to 4 mg/L; these target ELF exposures were also achieved in patients with moderate/severe renal impairment and patients with augmented renal clearance. These data, obtained from a robustly designed clinical trial that is unique in this therapeutic area, lend additional support to the clinical use of the 3 g q8h dosing regimen recently approved for HABP/VABP.^20^^,^^22–24^

## Supplementary Material

dkaa049_Supplementary_Data
