# Supplementary material for: Lung penetration, bronchopulmonary pharmacokinetic/pharmacodynamic profile and safety of 3 g of ceftolozane/tazobactam administered to ventilated, critically ill patients with pneumonia
Source: J Antimicrob Chemother. 2020 Mar 24;75(6):1546–53. doi: 10.1093/jac/dkaa049 (PMC7225904; doi:10.1093/jac/dkaa049)
Supplement: dkaa049_Supplementary_Data [file dkaa049_supplementary_data.zip › Supplementary_Data_I.docx]

**Supplementary data**

**Figure S1.** Individual and arithmetic mean ELF concentration-time profile for the last dose, with relevant minimum inhibitory concentration values shown as horizontal lines, among patients included in the ELF pharmacokinetic analyses (N=22), for (A) total ceftolozane and (B) total tazobactam.


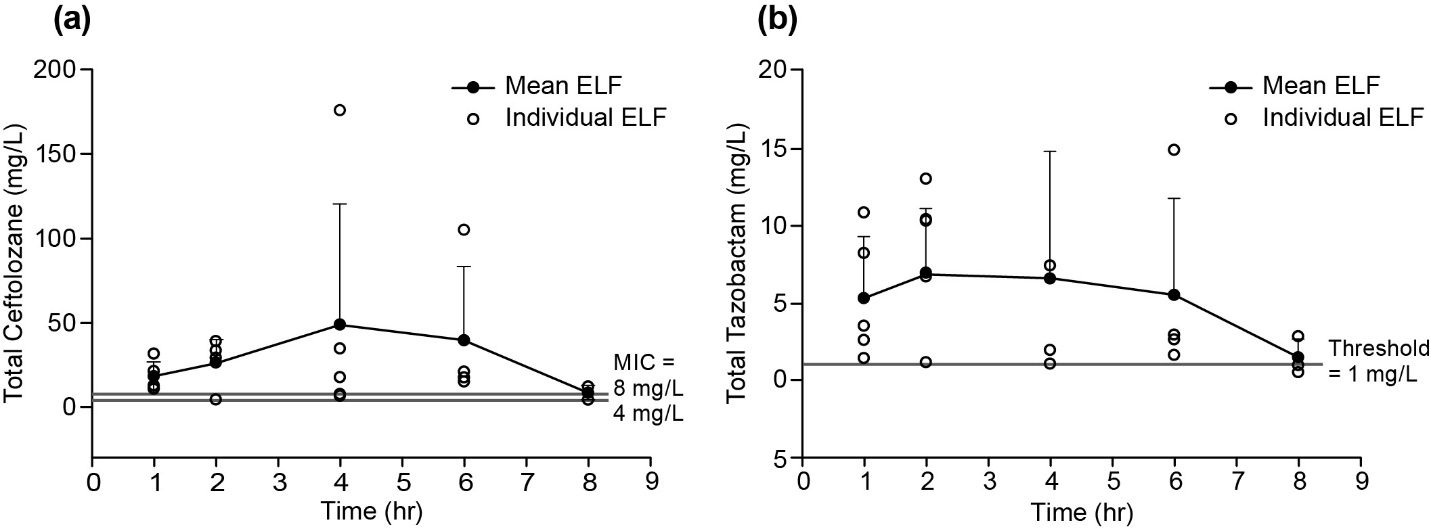


**Figure S2.** Average drug concentrations in patients with normal renal function (curves showing median concentrations along with 5^th^ and 95^th^ percentiles) compared to individual concentrations in patients with renal impairment (points labeled ‘Patient A’, ‘Patient B’, etc. show individual concentrations for each specific, dose-adjusted patient) for (A) unbound ceftolozane in plasma after first dose and last dose, (B) total ceftolozane in ELF after last dose, (C) unbound tazobactam in plasma after first dose and last dose, and (D) total tazobactam in ELF after last dose.


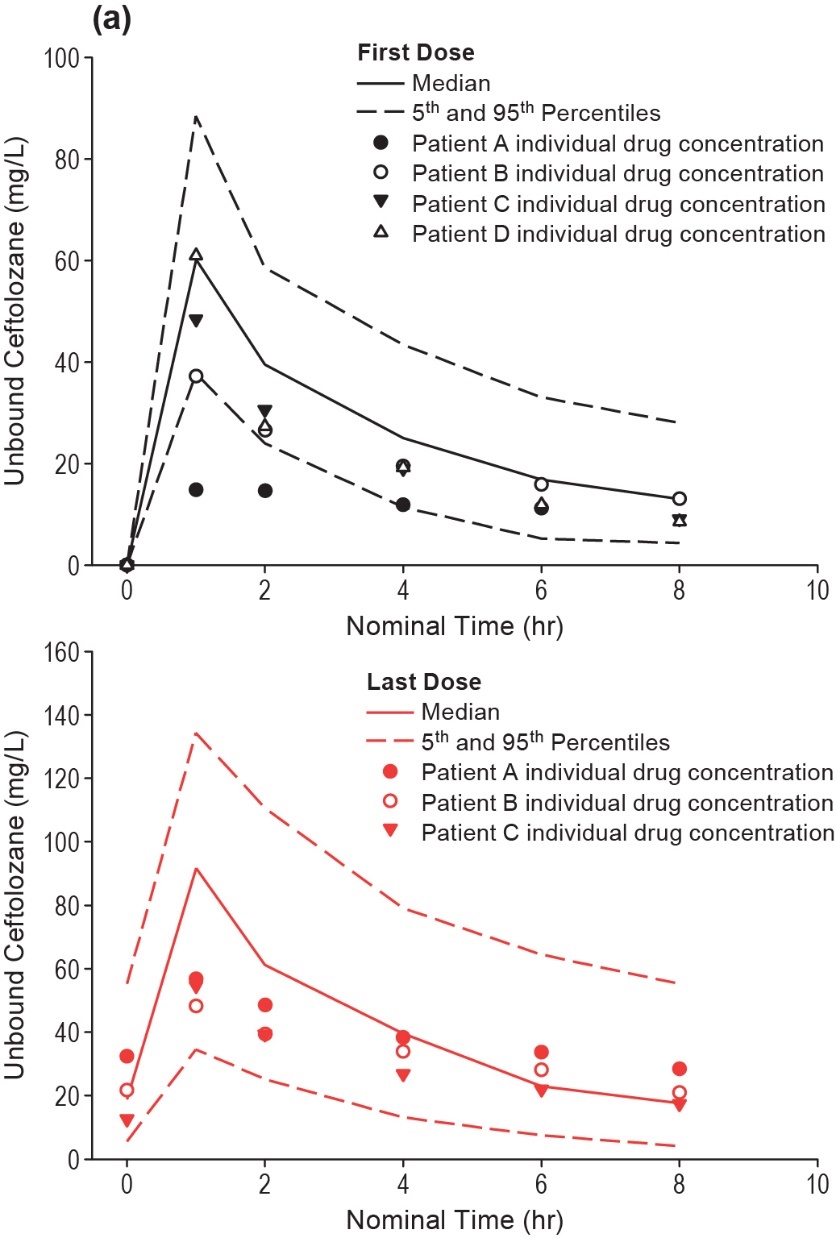


Note: 1 patient (Patient D) who was initially dose-adjusted to 1.5 g ceftolozane/tazobactam had sufficient renal function recovery to receive 3 g ceftolozane/tazobactam for doses 5 and 6; this patient was included into the calculation of median concentration for the last dose plot.


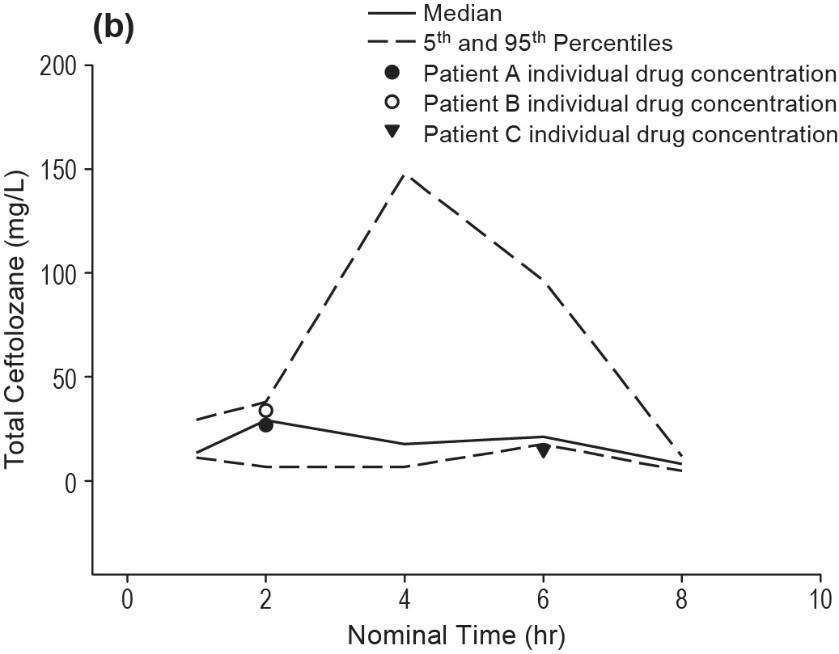


Note: 1 patient who was initially dose dose-adjusted to 1.5 g ceftolozane/tazobactam (and later had sufficient renal function recovery to receive 3 g ceftolozane/tazobactam for doses 5 and 6) was excluded from the ELF pharmacokinetic population entirely because of BAL urea concentrations falling below the lower limit of quantification.


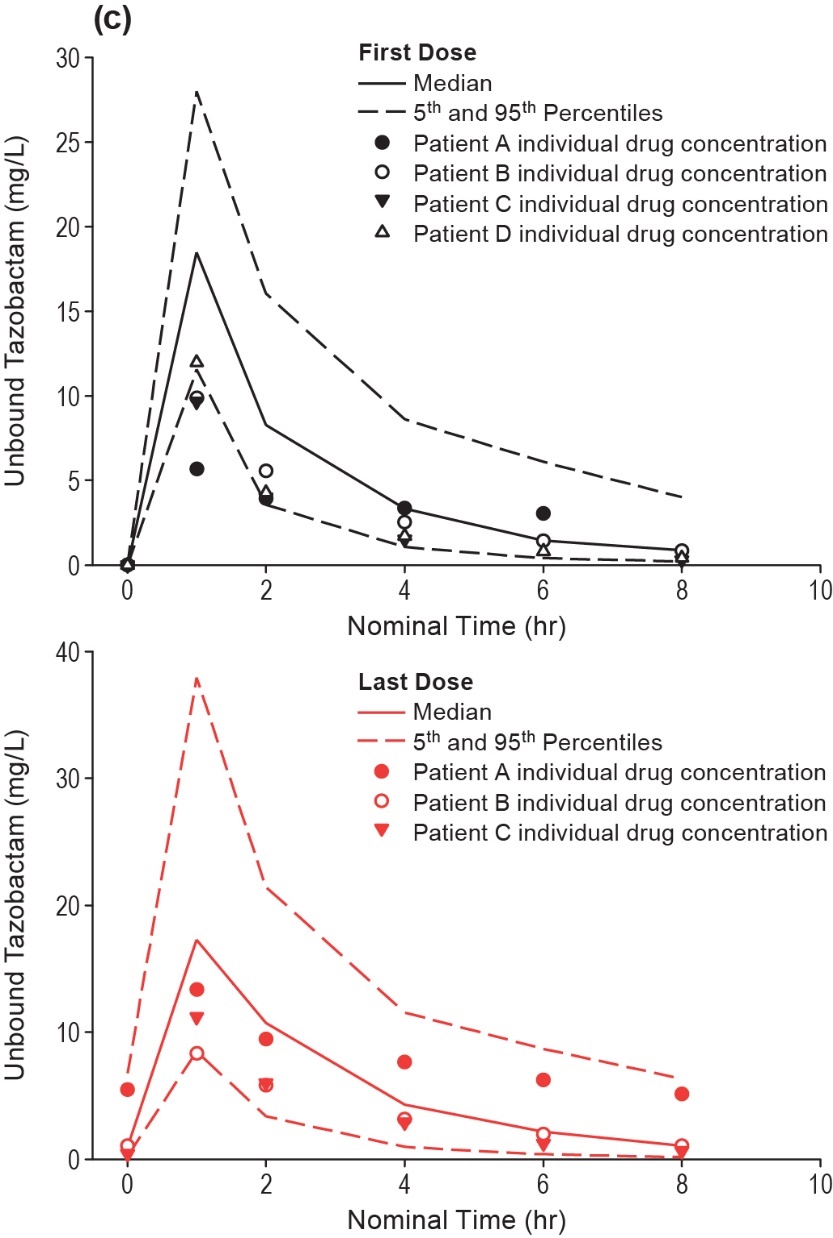


Note: 1 patient who was initially dose dose-adjusted to 1.5 g ceftolozane/tazobactam had sufficient renal function recovery to receive 3 g ceftolozane/tazobactam for doses 5 and 6; this patient was included into the calculation of median concentration for the last dose plot.

**
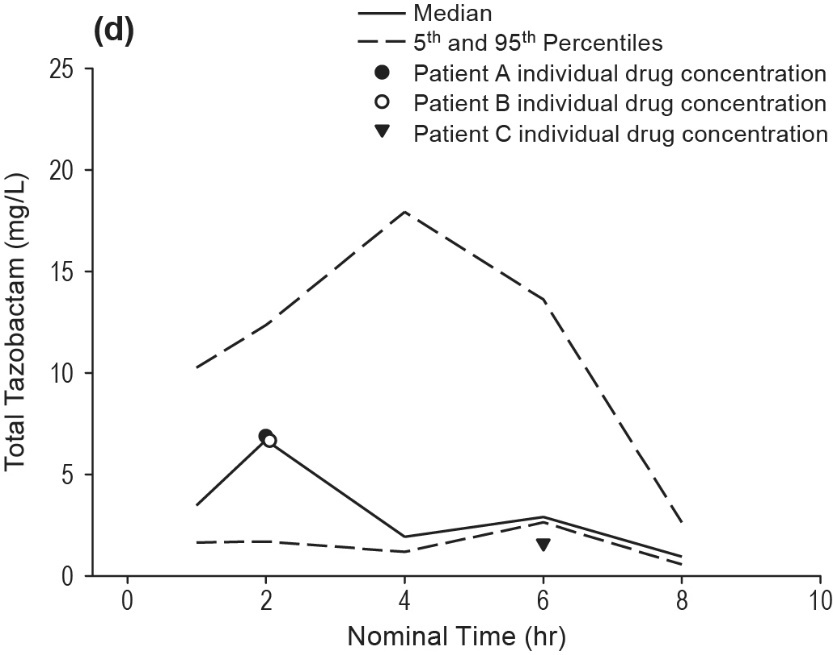
**

Note: 1 patient who was initially dose dose-adjusted to 1.5 g ceftolozane/tazobactam (and later had sufficient renal function recovery to receive 3 g ceftolozane/tazobactam for doses 5 and 6) was excluded from the ELF pharmacokinetic population entirely because of BAL urea concentrations falling below the lower limit of quantification.

**Table S1.** Number of patients in this study cohort who were in the safety population (i.e., received ≥1 dose of study drug), by country and investigational site (with primary site investigator)

|  | **Safety population**  **(N=26)** | **Independent ethics committee** |
| --- | --- | --- |
| **Belgium, n (%)** | **8 (30.7%)** |  |
| Ghent University Hospital, Ghent  Jan De Waele, MD | 7 (26.9%) | Commissie voor Medische  Ethiek, Universitair Ziekenhus  Gent  De Pintelaan 185  CXA- Gent, Belgium 9000 |
| University Hospital Leuven, Leuven  Joost Wauters, MD | 1 (3.8%) |  |
| **Spain, n (%)** | **3 (11.5%)** |  |
| University Hospital Clinic of Barcelona, Barcelona  Antoni Torres Marti, MD | 3 (11.5%) | CEIC Hospital Clinic I Provincial  C/Villarroel, 170  Barcelona Spain 08036 |
| **United States, n (%)** | **15 (57.6%)** |  |
| VA Hospital, San Juan, Puerto Rico  Onix Cantres-Fonseca, MD | 3 (11.5%) | VA Caribbean Healthcare System IRB  10 Casia Street  San Juan, Puerto Rico USA  00921 |
| Kentucky Lung Clinic, Hazard, KY  Firas A. Koura, MD | 2 (7.6%) | Appalachian Regional Healthcare, Inc., IRB  100 Airport Gardens Road  Hazard, KY USA 41701 |
| Hartford Hospital, Hartford, CT  David P. Nicolau, PharmD | 5 (19.2%) | Hartford Hospital Institutional  Review Board  80 Seymour Street  Hartford, CT USA 06102 |
| Henry Ford Hospital, Detroit MI  Katherine Reyes, MD | 1 (3.8%) | Henry Ford Health System  2799 W. Grand Blvd.  Detroit, MI USA 48202 |
| University of Tennessee Health Science Center, Memphis, TN  Joseph Swanson, PharmD | 2 (7.6%) | University of Tennessee Health  Science Center Institutional Review Board  910 Madison Ave., Suite 600  Memphis, TN USA 38163 |
| Northwestern Medical Center, Chicago, IL  Richard Wunderink, MD | 2 (7.6%) | Northwestern University;  Biomedical IRB, Institutional  Review Board Office  750 North Lake Shore Drive,  Suite 700  Chicago, IL USA 60611 |

**Table S2.** Geometric mean (range) for relevant PK/PD measures in plasma and ELF,^a^ for both ceftolozane and tazobactam, following administration of 3 g ceftolozane/tazobactam q8h (this dosing regimen was adjusted for renal function)

| **Ceftolozane** | | | |
| --- | --- | --- | --- |
| **PK/PD measure** | **Plasma** | | **ELF** |
|  | **First Dose** | **Last Dose** | **Last Dose** |
|  | **(N=25)** | **(N=24)** | **(N=22)** |
| %*f*T > 4 mg/L | 97.9 (85.6-99.5) | 99.9 (98.1-100) | 100^a^ |
| %*f*T > 8 mg/L | 88.6 (55.0-99.1) | 95.5 (63.8-100) | 100^a^ |
| **Tazobactam** | | | |
| **PK/PD measure** | **Plasma** | | **ELF** |
|  | **First Dose** | **Last Dose** | **Last Dose** |
|  | **(N=25)** | **(N=24)** | **(N=22)** |
| %*f*T > Threshold of 1 mg/L | 77.5 (45.1-99.5) | 82.4 (35.1-100) | 100^a^ |

%*f*T > MIC, percentage of time the free (non-protein-bound) ceftolozane concentration is above the minimum inhibitory concentration needed for bactericidal activity. %*f*T > Threshold, percentage of time the free (non-protein-bound) tazobactam concentration is above the threshold concentration needed for inhibition of susceptible β-lactamases.

^a^No range is presented for ELF, since PK/PD target attainment in ELF was estimated using a composite profile.

**Table S3.** Summary of patients with treatment-emergent adverse events in the safety population (N=26)

|  | **3 g ceftolozane/ tazobactam**  **(N=21)** | **1.5 g ceftolozane/ tazobactam**  **(N=4)** | **750 mg ceftolozane/ tazobactam**  **(N=1)** |
| --- | --- | --- | --- |
| ≥ 1 AE, n (%) | 14 (66.7%) | 1 (25.0%) | 1 (100.0%) |
| Mild, n (%) | 7 (33.3%) | 1 (25.0%) | 1 (100.0%) |
| Moderate, n (%) | 7 (33.3%) | 0 | 0 |
| Severe, n (%) | 0 | 0 | 0 |
| ≥ 1 serious AE, n (%) | 0 | 0 | 0 |
| ≥ 1 treatment-related AE, n (%) | 2 (9.5%) | 0 | 0 |

**Table S4.** Summary of all treatment-emergent adverse events in the safety population (N=26) of this study cohort, by preferred term and frequency, regardless of dose administered. Patients could have had more than one adverse event, including more than one per category

| **Treatment-emergent adverse events** | **Safety Population** |
| --- | --- |
|  | **(N=26)** |
| Patients with ≥1 adverse event, n (%) | 16 (61.5) |
| Patients with no adverse event, n (%) | 10 (38.5) |
| **Blood and lymphatic system disorders, n (%)** | **3 (11.5)** |
| Anemia | 2 (7.7) |
| Leukocytosis | 1 (3.8) |
| Thrombocytopenia | 1 (3.8) |
| **Cardiac disorders, n (%)** | **3 (11.5)** |
| Atrial fibrillation | 1 (3.8) |
| Cyanosis | 1 (3.8) |
| Tachycardia | 1 (3.8) |
| Ventricular tachycardia | 1 (3.8) |
| **Gastrointestinal disorders, n (%)** | **6 (23.1)** |
| Constipation | 1 (3.8) |
| Diarrhea | 4 (15.4) |
| Nausea | 1 (3.8) |
| Vomiting | 1 (3.8) |
| **General disorders & administration site conditions, n (%)** | **1 (3.8)** |
| Pyrexia | 1 (3.8) |
| **Infections and infestations, n (%)** | **1 (3.8)** |
| Device related infection | 1 (3.8) |
| **Injury, poisoning and procedural complications, n (%)** | **1 (3.8)** |
| Mechanical ventilation complication | 1 (3.8) |
| **Investigations, n (%)** | **1 (3.8)** |
| Hepatic enzyme increased | 1 (3.8) |
| **Metabolism and nutrition disorders, n (%)** | **3 (11.5)** |
| Hyperglycemia | 2 (7.7) |
| Hypoglycemia | 1 (3.8) |
| Hypokalemia | 1 (3.8) |
| Vitamin D deficiency | 1 (3.8) |
| **Musculoskeletal and connective tissue disorders, n (%)** | **1 (3.8)** |
| Limb deformity | 1 (3.8) |
| **Nervous system disorders, n (%)** | **1 (3.8)** |
| Autonomic nervous system imbalance | 1 (3.8) |
| Intracranial pressure increased | 1 (3.8) |
| **Psychiatric disorders, n (%)** | **1 (3.8)** |
| Agitation | 1 (3.8) |
| **Renal and urinary disorders, n (%)** | **1 (3.8)** |
| Hematuria | 1 (3.8) |
| **Respiratory, thoracic and mediastinal disorders, n (%)** | **6 (23.1)** |
| Bronchospasm | 1 (3.8) |
| Hemoptysis | 1 (3.8) |
| Hyperventilation | 1 (3.8) |
| Pleural effusion | 1 (3.8) |
| Pulmonary edema | 2 (7.7) |
| Tachypnea | 1 (3.8) |
| **Vascular disorders, n (%)** | **6 (23.1)** |
| Hypertension | 4 (15.4) |
| Hypotension | 3 (11.5) |
| Treatment-emergent adverse events: adverse events that occurred from the time of first dose of study drug through the last study evaluation or pre-existing adverse events that were aggravated in severity and/or frequency during the dosing period. | |
