# Supplementary material for: Lung penetration, bronchopulmonary pharmacokinetic/pharmacodynamic profile and safety of 3 g of ceftolozane/tazobactam administered to ventilated, critically ill patients with pneumonia
Source: J Antimicrob Chemother. 2020 Mar 24;75(6):1546–53. doi: 10.1093/jac/dkaa049 (PMC7225904; doi:10.1093/jac/dkaa049)
Supplement: dkaa049_Supplementary_Data [file dkaa049_supplementary_data.zip › Supplementary_Data_II.pdf]

Merck & Co., Inc. policy on posting of redacted study protocols on journal websites is described in Guidelines for Publication of Clinical Trials in the Scientific Literature on the www.merck.com website.

For publicly posted protocols, the Company redacts content that contains proprietary and private information.

This report may include approved and non-approved uses, formulations, or treatment regimens. The results reported may not reflect the overall profile of a product. Before prescribing any product mentioned in this report, healthcare professionals should consult local prescribing information for the product approved in their country.

Copyright © 2020 Merck Sharp & Dohme Corp., a subsidiary of Merck & Co., Inc. All Rights Reserved. Not for regulatory or commercial use.

CEFTOLOZANE/TAZOBACTAM

CXA-ICU-14-01

A PHASE 1, PROSPECTIVE, MULTI-CENTER, OPEN-LABEL STUDY TO ASSESS THE PLASMA PHARMACOKINETICS AND LUNG PENETRATION OF INTRAVENOUS (IV) CEFTOLOZANE/TAZOBACTAM IN CRITICALLY ILL PATIENTS

**Study Drug**:	Ceftolozane/tazobactam
**IND Number:**	104, 490
**Protocol Number**:	CXA-ICU-14-01
**Sponsor**:	Cubist Pharmaceuticals LLC,<br>an indirect wholly-owned subsidiary of<br>Merck Sharp & Dohme Corp.<br>65 Hayden Ave<br>Lexington, MA 02421<br>PPD
**Dose Form**:	Intravenous
**Medical Monitor**:	PPD
**Version:**	3.0
**Date:**	04 November 2015

STATEMENT OF CONFIDENTIALITY

The confidential information in the following document is provided to you as an investigator, potential investigator, or consultant, for review by you, your staff, and applicable institutional review board(s). It is understood that the information will not be disclosed to others without written authorization from Cubist Pharmaceuticals LLC, an indirect wholly-owned subsidiary of Merck Sharp & Dohme Corp., except to the extent necessary to obtain informed consent from those persons to whom the drug may be administered.

PROTOCOL APPROVAL

**Protocol Title:**	A Phase 1, Prospective, Multi-center, Open-label Study to Assess the Plasma Pharmacokinetics and Lung Penetration of Intravenous (IV) Ceftolozane/tazobactam in Critically Ill Patients
**Protocol Number:**	CXA-ICU-14-01
**Protocol Date:**	04 November 2015

PPD

[redacted]

04-Nov-2015

Date

INVESTIGATOR'S AGREEMENT

I have received and read the Investigator's Brochure for ceftolozane/tazobactam. I have read the CXA-ICU-14-01 protocol and agree to conduct the study as outlined. I agree to maintain the confidentiality of all information received or developed in connection with this protocol.

____

Printed Name of Investigator

____

Signature of Investigator

____

Date

1. SYNOPSIS

**Name of Sponsor/Company:** Cubist Pharmaceuticals LLC, an indirect wholly-owned subsidiary of Merck Sharp & Dohme Corp. (Cubist)
**Name of Investigational Product:** Ceftolozane/tazobactam
**Name of Active Ingredient:** Ceftolozane/tazobactam
**Title of Study:** A Phase 1, Prospective, Multicenter, Open-label Study to Assess the Plasma Pharmacokinetics and Lung Penetration of Intravenous (IV) Ceftolozane/tazobactam in Critically Ill Patients
**Phase of development:** 1
**Objectives:**<br>**Primary:**<br>• To characterize the plasma pharmacokinetics (PK) and intrapulmonary penetration, as measured by concentrations in the ELF, of ceftolozane/tazobactam in mechanically ventilated patients concurrently receiving standard antibiotic therapy for proven or suspected pneumonia<br>**Secondary:**<br>• To characterize the plasma PK of ceftolozane/tazobactam after a single dose in critically ill patients with a creatinine clearance (CL_{CR}) ≥ 180 mL/min (as calculated by the Cockcroft-Gault equation)<br>• To assess the safety and tolerability of ceftolozane/tazobactam<br>**Other:**<br>• To assess the effect of inflammatory response, as measured by inflammatory markers (C-reactive protein [CRP] and albumin), on the PK and intrapulmonary penetration of ceftolozane/tazobactam<br>• To explore the correlation between baseline measured CL_{CR} (from the 8-hour urine collection) and baseline estimated CL_{CR} (as calculated from the Cockcroft-Gault equation)
**Study Rationale:**<br>Nosocomial pneumonia is one of the most common hospital-acquired infections, can be life-threatening, and often occurs in patients on mechanical ventilation. Gram-negative bacteria are commonly implicated in cases of nosocomial pneumonia, most notably *Pseudomonas aeruginosa* (including multi-drug resistant strains), Enterobacteriaceae (including extended-spectrum β-lactamase [ESBL]-producing strains), and *Acinetobacter baumannii* [1, 2]. The choice of appropriate empiric antibiotic therapy for nosocomial pneumonia is typically based on local resistance epidemiology data. The dose is often based on the PK and pharmacodynamics (PD) of the antibiotic therapy, including concentrations at the site of infection. Despite limited data correlating clinical outcome and concentration at the site of infection in nosocomial pneumonia where target pathogens are located (ie, the epithelial lining fluid [ELF]), achieving clinically significant concentrations in the ELF remains important in dose selection [3].<br>A study in healthy volunteers demonstrated that ceftolozane/tazobactam penetrates well into the intrapulmonary space [4]. Extrapolation of these results to patients with infected lungs is challenging due to the multiple factors (e.g., capillary leak, fluid loading, and altered renal function) that may distort the normal PK profile. Due to the potential for local inflammatory response to alter study drug penetration into the lung, plasma and BAL samples will be evaluated for ceftolozane/tazobactam

concentrations in patients with pneumonia. In addition, modifications of albumin concentration in ELF are associated with the presence of inflammatory processes in the lower respiratory tract, and CRP is a well-known biochemical marker of inflammation [5, 6]. Therefore, CRP and albumin will also be assessed as biomarkers to identify any potential association between the patients' inflammatory response and intrapulmonary penetration of ceftolozane/tazobactam. Collectively, data generated will help understand how nosocomial pneumonia pathophysiology affects the PK of ceftolozane/tazobactam to provide critical information for the development of ceftolozane/tazobactam in nosocomial pneumonia.

Augmented renal clearance (ARC) has been reported in critically ill patients, can result in sub-therapeutic plasma concentrations of renally excreted antibiotics, and has been associated with a higher incidence of therapeutic failure and worse clinical outcomes [7, 8]. Ceftolozane/tazobactam is primarily renally eliminated, therefore maximizing time above the minimum inhibitory concentration (T>MIC) may prove challenging in this patient population. The prevalence of ARC has not been consistently defined in the literature, varying from CL_{CR} > 120 to 160 mL/min [9, 10]. In adult Phase 2 and 3 complicated urinary tract infection (cUTI) and complicated intra-abdominal infection (cIAI) studies with ceftolozane/tazobactam, approximately 2-6% of patients exhibited CL_{CR} greater than 180 mL/min. To supplement this existing PK database for ceftolozane/tazobactam, this study will characterize the plasma concentrations of ceftolozane/tazobactam in critically ill patients with a $CL_{CR} \geq 180$ mL/min. This information can be used to optimize the dosage regimen of ceftolozane/tazobactam in those with augmented renal clearance.

Methodology:

This is a Phase 1, prospective, multicenter, non-comparative, open-label study to characterize the plasma pharmacokinetics and intrapulmonary penetration of ceftolozane/tazobactam in two groups of patients.

> **Group 1:** approximately 25 ventilated patients with proven or suspected pneumonia receiving concurrent standard antibiotic therapy. Within Group 1, efforts will be made to enroll approximately 5 patients with a $CL_{CR} \geq 150$ mL/min (as calculated by the Cockcroft-Gault equation).
> **Group 2:** 8-10 critically ill patients with $CL_{CR} \geq 180$ mL/min (as calculated by the Cockcroft-Gault equation).

Baseline (screening) procedures in all patients, including medical and surgical history, demographics, vital signs, serum pregnancy test (as applicable), and clinical laboratory tests (hematology, chemistry, coagulation) will be performed within 24 hours prior to dosing.

Patients in Group 1 will receive ceftolozane/tazobactam as a 60-minute intravenous infusion every 8 hours for a total of 4-6 doses. Patients with severe renal impairment (CL_{CR} 15 – 29 mL/min) at baseline must receive a total of 6 doses. Plasma PK sampling will occur at the following time points: 0 (pre-dose), 1, 2, 4, 6, and 8 hours after start of first and last (4th, 5th, or 6th) infusions.

Every patient in Group 1 will undergo only one bronchoalveolar lavage (BAL) procedure at one of the following five time points: 1, 2, 4, 6, or 8 hours post start of infusion after the final (4th, 5th, or 6th) dose. Blood samples collected at the time of BAL sampling will also be tested for inflammatory markers. In the event that a patient in Group 1 undergoes a BAL procedure as part of standard of care (at any time point post-dose during the study), an additional sample may be collected for PK analysis. The timing of each BAL sample will be assigned. There will be approximately 5 patients per BAL time point in Group 1. The BAL and plasma data points will be used to construct a concentration-time profile.

Patients in Group 2 will receive a single dose of ceftolozane/tazobactam (3 g) as a 60-minute intravenous infusion. Blood samples will be collected to characterize plasma PK at the following time

points: 0 (pre-dose), 1, 2, 4, 6, and 8 hours after the start of infusion. Patients in Group 2 will not undergo a BAL procedure.

In all patients who are catheterized, 8- hour urine collections will be obtained for direct measurement of CL_{CR} and an exploratory analysis will be performed to compare baseline measured CL_{CR} with baseline estimated CL_{CR} (as calculated by the Cockcroft-Gault equation).

An interim PK analysis will be performed after 3 BAL samples at each scheduled sampling time point are available. Enrollment in Group 1 may stop early if the estimated overall inter-individual variability is not higher than, or the estimated minimum penetration ratio is not lower than, that observed in the previous ELF study in healthy volunteers (CXA-ELF-10-03). Enrollment in Group 1 may continue beyond 25 patients if the penetration ratio cannot be reliably estimated.

All patients will be followed for safety for 24-48 hours following administration of the final dose of study drug.

Number of patients (planned):

Group 1: Approximately 25 mechanically ventilated patients concurrently receiving standard antibiotic therapy for proven or suspected pneumonia.

Group 2: 8 – 10 critically ill patients with CL_{CR} ≥180 mL/min.

Inclusion Criteria:

To be eligible for enrollment, a patient must fulfill all of the following inclusion criteria:

1. Provide written informed consent prior to any study-related procedure not part of normal medical care. If the patient is unable to do so, local country laws and institution specific guidelines and requirements in place for obtaining informed consent should be met. A legally acceptable representative may provide consent, provided this is approved by local country and institution specific guidelines. If a patient is able to provide consent while still in the study and per the Investigator’s judgment the patient is able to read, assess, understand and make his/her own decision to participate in the trial, the patient must agree to continue study participation and the patient may be re-consented, if required by local country and institution specific guidelines;
2. If female, patient must not be pregnant or nursing, and is either:
    a. Not of childbearing potential, defined as postmenopausal for at least 1 year or surgically sterile due to bilateral tubal ligation, bilateral oophorectomy, or hysterectomy; or
    b. Of childbearing potential and:
        - Is practicing an effective method of contraception (e.g., oral/parenteral contraceptives or a barrier method) and for at least 1 month prior to baseline assessments, or
        - Has a vasectomized partner, or
        - Is currently abstinent from sexual intercourse.

    Patients must be willing to practice the chosen contraceptive method or remain abstinent during the conduct of the study and for at least 30 days after last dose of study medication.
3. Non-vasectomized males are required to practice effective birth control methods (e.g., abstinence, use of a condom or use of other barrier device) during the conduct of the study and for at least 30 days after last dose of study medication;
4. Patients in Group 1 must meet the following criteria:
    a. Male or female patients age 18 years or older;
    b. Intubated and on mechanical ventilation for at least 24 hours prior to time of enrollment (includes patients with tracheostomy who are mechanically ventilated) and

anticipated to be on mechanical ventilation for at least 8 hours following the final dose of study drug;

c. Proven or suspected bacterial pneumonia, as confirmed by the presence of **at least one** of the following clinical signs and symptoms within the past 48 hours:
   - Documented fever (oral, rectal, tympanic, or core temperature > 38.5°C)
   - Hypothermia (oral, rectal, tympanic, or core temperature < 35.0°C)
   - An elevated white blood cell (WBC) count ≥12,000 cells/mm3
   - Radiological findings suggestive of bacterial pneumonia

d. Receiving antibiotic therapy for proven or suspected bacterial pneumonia at the time of enrollment and expected to continue on antibiotic therapy while in the study

5. Patients in Group 2 must meet the following criteria:
   a. Male or female aged 18 – 54 years;
   b. APACHE II score between 12 and 35, inclusive;
   c. Creatinine clearance (CL_{CR}) ≥180 mL/min (as calculated by the Cockcroft-Gault equation using actual body weight) within 24 hours of dosing;
   d. Documented infection or presumed infection as confirmed by the presence of **at least** one of the following criteria within the past 72 hours:
      - Documented fever (oral, rectal, tympanic, or core temperature > 38.5°C)
      - Hypothermia (oral, rectal, tympanic, or core temperature < 35.0°C)
      - An elevated white blood cell (WBC) count ≥12,000 cells/mm3

Exclusion Criteria:

To be eligible for enrollment, a patient must not meet any of the following exclusion criteria:

1. Has a documented history of any moderate or severe hypersensitivity or allergic reaction to any β-lactam antibacterial (a history of a mild rash followed by uneventful re-exposure is not a contraindication to enrollment);
2. Hemoglobin < 7 gm/dL at baseline;
3. Prior (within 24 hours of first dose of study drug) or concomitant receipt of piperacillin/tazobactam, probenecid, or ceftolozane/tazobactam (non-study use);
4. Any rapidly-progressing disease or immediately life-threatening illness (defined as imminent death within 48 hours in the opinion of the Investigator);
5. Any condition or circumstance that, in the opinion of the Investigator, would compromise the safety of the patient or the quality of study data;
6. Planned or prior participation in any interventional drug study within the last 30 days;
7. Patients in Group 1 must not meet any of the following criteria:
   a. Receipt of effective systemic antibiotic therapy for the treatment of proven or suspected bacterial pneumonia for more than 72 hours prior to start of the first dose of study drug

      Note: If signs and/or symptoms of bacterial pneumonia have not improved despite >72 hours of the antibacterial regimen, a patient is still eligible. This will require discussion with and approval from a medical monitor

   b. Any of the following diagnoses or conditions that may interfere with the PK assessment/interpretation:
      - Cystic fibrosis, acute exacerbation of chronic bronchitis or obstructive airway disease, chronic severe respiratory disease , or active pulmonary tuberculosis
      - Full thickness burns (greater than 15% of total body surface area),
      - Lung transplant recipient or donor,

- Any condition or situation where bronchoscopy is not advisable;

c. End-stage renal disease defined as a CL_{CR} < 15 mL/min (as calculated by the Cockcroft-Gault equation using actual body weight), OR requirement for continuous renal replacement therapy or hemodialysis.

Investigational product, dosage and mode of administration:

Dosing of ceftolozane/tazobactam in Group 1:

Renal Function	Dose	Infusion Frequency	Duration of Infusion
CL_{CR} > 50 mL/min	3 g IV	q8h (± 1 hour)	60 ± 10 min infusion
CL_{CR} 30 – 50 mL/min	1.5 g IV	q8h (± 1 hour)	60 ± 10 min infusion
CL_{CR} 15 – 29 mL/min	750 mg IV	q8h (± 1 hour)	60 ± 10 min infusion

The dose of ceftolozane/tazobactam should be adjusted accordingly based on post-baseline assessments of creatinine clearance (i.e. calculated or measured).

Dosing of ceftolozane/tazobactam in Group 2: 3 g IV as a 60 ± 10 minute infusion

Duration of treatment:

All patients will be screened for eligibility within 24 hours prior to study drug administration and monitored 24 hours after last dose of study drug.

Duration of therapy in Group 1 will be 2 days (4-6 doses of ceftolozane/tazobactam).

Group 2 will only receive a single dose of ceftolozane/tazobactam.

Criteria for evaluation:

Analysis populations will consist of:

- *Pharmacokinetic (PK) population:*

  Group1: The PK population to evaluate ELF penetration will consist of patients who receive at least 4 doses (6 doses for patients with severe renal impairment [CL_{CR} 15 – 29 mL/min] at baseline) of ceftolozane/tazobactam and who provide at least one serial blood sample and one BAL sample post-completion of infusion.

  Group 2: The PK population will consist of subjects who receive 1 dose of ceftolozane/tazobactam and who provide at least 3 serial blood samples post-completion of infusion, inclusive of C_{max}, to be able to estimate the area under the curve (AUC).

  The criteria for excluding a subject from the final PK analysis will be detailed in the Pharmacokinetics and Statistical Analysis Plan.

- *Safety population:* All patients who receive study medication (including partial doses).

Pharmacokinetics:

The primary endpoint is the characterization of plasma PK (Group 1 and Group 2) and intrapulmonary penetration that includes determination of the ELF to plasma ratio of ceftolozane and tazobactam concentrations (Group 1).

Plasma PK data will be summarized by descriptive statistics, including mean, standard deviation (SD), median, coefficient of variation (CV%), minimum, maximum, geometric mean, and geometric CV, and presented for each time point by group for plasma concentrations of ceftolozane and tazobactam. Individual and mean ceftolozane and tazobactam plasma concentration-time profiles will be plotted.

The following plasma PK parameters will be determined as allowed by the data: maximum plasma concentration (C_{max}), time to C_{max} (T_{max}), concentration at last sampling point (C_{last}), time of last sampling point (T_{last}), area under the plasma concentration-time curve from 0 to last sample collected ($AUC_{0\text{-}last}$), area under the plasma concentration-time curve from 0 to infinity ($AUC_{0\text{-}\infty}$), elimination half-life ($t_{1/2}$), volume of distribution at steady state (V_{ss}), and plasma clearance (CL).

The ELF concentration at each time point will be summarized by descriptive statistics, including mean, SD, median, coefficient of variation (CV%), minimum, maximum, geometric mean, and geometric CV, and presented for each time point by ELF concentrations of ceftolozane and tazobactam. Mean ceftolozane and tazobactam ELF concentration-time profiles will be plotted. Exploratory graphical analysis comparing ELF and plasma concentration may be done.

From the composite (geometric mean) ELF concentration-time profile, the following ELF PK parameters will be determined as allowed by the data: maximum ELF concentration (C_{max}), time to ELF C_{max} (T_{max}), ELF concentration at last sampling point (C_{last}), time of last BAL sampling point (T_{last}), area under the composite mean ELF concentration-time curve from 0 to last sample collected ($AUC_{0\text{-}last}$), area under the composite mean ELF concentration-time curve from 0 to infinity ($AUC_{0\text{-}\infty}$), ELF elimination half-life ($t_{1/2}$), and percent penetration into ELF (calculated as the ratio of $AUC_{0\text{-}tELF}$ and mean $AUC_{0\text{-}tPlasma}$). In addition, ELF-to-plasma concentration ratio at each time point (C_{tELF}: $C_{tPlasma}$) and associated summary statistics will be included.

If data allow, a plasma-ELF population PK model will be used to further evaluate the potential target attainment in patients with augmented renal clearance. The plasma and ELF PK data from Group 1 will be used with plasma PK data from Group 2 to predict ceftolozane/tazobactam lung exposure in patients with augmented renal clearance. The population PK plan and results, if applicable, will be documented separately.

Pharmacokinetics/Pharmacodynamics:

Group 1: BAL and blood samples collected at the time of BAL sampling will also be tested for inflammatory markers (CRP and albumin) to evaluate the potential correlation between these inflammatory markers and ELF penetration of ceftolozane/tazobactam. Cytology counts will also be performed on BAL samples.

Safety:

This study will assess the safety and tolerability of IV ceftolozane/tazobactam in critically ill patients.

Safety and tolerability will be evaluated by examining the incidence, severity, and type of adverse events, changes in clinical laboratory tests, and changes from baseline in vital signs.

The type and incidence of all adverse events and serious adverse events while on study drug through last study contact will be tabulated. Laboratory data will be summarized by type of laboratory test. Descriptive statistics of laboratory values, vital signs and changes from baseline will be summarized.

Statistical Methods:

No formal statistical analysis is planned. Statistical methods are primarily descriptive in nature and will be used to guide decisions as to the clinical relevance of findings.

Safety analyses will include a summarization of AEs and tabulation of changes from baseline in clinical laboratory data and vital signs.

2. TABLE OF CONTENTS, LIST OF TABLES, AND LIST OF FIGURES

TITLE PAGE 1
1. SYNOPSIS 4
2. TABLE OF CONTENTS, LIST OF TABLES, AND LIST OF FIGURES 10
3. LIST OF ABBREVIATIONS AND DEFINITIONS OF TERMS 15
4. INTRODUCTION 18
4.1. Ceftolozane/tazobactam 18
4.2. Non-Clinical Experience 18
4.3. Clinical Experience with Ceftolozane/tazobactam 19
4.3.1. Pharmacokinetics 19
4.3.2. Safety 20
4.4. Rationale 21
4.4.1. Study Rationale 21
4.4.2. Dose Rationale 22
5. TRIAL OBJECTIVES AND PURPOSE 23
5.1. Primary Objective 23
5.2. Secondary Objectives 23
5.3. Other Objectives 23
6. INVESTIGATIONAL PLAN 24
6.1. Overall Study Design 24
6.1.1. Dosing and the Interim PK Assessment 25
6.2. Study Visits 25
6.3. Study Procedures 26
6.3.1. Clinical Assessments 26
6.3.2. Laboratory Assessments 26
6.3.3. Pharmacokinetic and Inflammatory Markers Sampling 27
6.3.3.1. Blood Sampling 27
6.3.3.2. BAL Sampling (Group 1) 27
6.4. Number of Patients 27
6.5. Treatment Assignment 27

6.6. Criteria for Study Termination 28
7. SELECTION AND WITHDRAWAL OF PATIENTS 31
7.1. Patient Inclusion Criteria 31
7.2. Patient Exclusion Criteria 32
7.3. Patient Withdrawal Criteria 33
7.4. Replacement of Patients 33
8. TREATMENT OF PATIENTS 34
8.1. Description of Study Drug 34
8.2. Concomitant Medications 34
8.3. Patient Identification and Numbering 34
8.4. BAL Assignment (Group 1) 34
9. STUDY DRUG MATERIALS AND MANAGEMENT 35
9.1. Study Drug 35
9.2. Study Drug Packaging, Labeling and Storage 35
9.3. Study Drug Preparation 35
9.4. Administration 35
9.5. Study Drug Accountability 35
10. PHARMACOKINETIC ASSESSMENTS 36
10.1. Blood Sample Collection 36
10.2. BAL Specimen Collection (Group 1) 36
10.3. Sample Handling and Processing 37
10.3.1. Blood Sample Handling and Processing 37
10.3.2. BAL Sample Handling and Processing 37
11. ASSESSMENT OF SAFETY 39
11.1. Definition of Adverse Events 39
11.1.1. Adverse Events 39
11.1.2. Serious Adverse Event 39
11.1.3. Overdose 40
11.2. Monitoring of Adverse Events 40
11.3. Monitoring of Laboratory Assessments 40
11.4. Assessment of Adverse Events 40
11.4.1. Assessment of Severity 40
11.4.2. Assessment of Causality 41

11.4.3. Reference Safety Information for the Assessment of Expectedness of AEs 42
11.5. Reporting Safety Observations by the Investigator to the Sponsor 42
11.5.1. Reporting of Nonserious AEs 42
11.5.2. Reporting of Drug Exposure During Pregnancy 42
11.5.3. Reporting of Expedited Safety Observations by the Investigator 42
11.6. Expedited Reporting by the Sponsor to the FDA 43
11.7. Safety Notifications by the Sponsor to the Investigator 43
12. STATISTICAL CONSIDERATIONS 44
12.1. Data Collection, Processing and Reporting 44
12.2. Populations for Analysis 44
12.2.1. PK Population 44
12.2.2. Safety Population 45
12.2.3. Endpoints 45
12.2.3.1. Primary Endpoint 45
12.2.3.2. Secondary Endpoint 45
12.2.4. Interim Analysis 45
13. PHARMACOKINETIC ANALYSIS PLAN 46
13.1.1. Pharmacokinetic Parameters 46
13.1.2. Below Limit of Quantification Values/Missing Values 46
13.1.3. Pharmacokinetic/Pharmacodynamic Analysis (Group 1) 46
13.2. Presentation of Data 47
13.3. PK Data Reporting 47
13.4. Data Displays 47
14. STATISTICAL ANALYSIS PLAN 48
14.1. General Methodology 48
14.1.1. Baseline Definitions 48
14.1.2. Multiple Comparisons/Multiplicity 48
14.1.3. Computing Environment 48
14.1.4. Patient Disposition 48
14.1.5. Demographics and Baseline Data 48
14.1.6. Study Drug Exposure 49
14.1.7. Secondary Endpoint Analysis 49
14.1.8. Adverse Events 49

14.1.9. Laboratory Data 50
14.1.10. Vital Signs 50
14.1.11. Concomitant Medications 50
14.1.12. Sample Size and Power Considerations 50
14.1.13. Evaluation Criteria 51
15. DIRECT ACCESS TO SOURCE DATA/DOCUMENTS 52
15.1. Study Monitoring 52
15.1.1. Protocol Deviations/Amendments 52
15.1.2. Discontinuation of the Study 53
15.2. Audits and Inspections 53
15.3. Institutional Review Board (IRB) 53
16. QUALITY CONTROL AND QUALITY ASSURANCE 54
17. ETHICS 55
17.1. Ethics Review 55
17.2. Ethical Conduct of the Study 55
17.3. Written Informed Consent 55
18. DATA HANDLING AND RECORDKEEPING 56
18.1. Inspection of Records 56
18.2. Retention of Records 56
19. PUBLICATION POLICY 57
20. LIST OF REFERENCES 58
21. APPENDICES 59
21.1. Appendix A: Acute Physiology and Chronic Health Evaluation (APACHE) II Severity of Disease Classification System 59

LIST OF TABLES

Table 1: Ceftolozane/tazobactam Dosing in Group 1 25
Table 2: Schedule of Assessments – Group 1 29
Table 3: Schedule of Assessments – Group 2 30

3. LIST OF ABBREVIATIONS AND DEFINITIONS OF TERMS

Abbreviation or Specialist Term	Explanation
AE	Adverse event
ALT (SGPT)	Alanine aminotransferase
APACHE II	Acute Physiology and Chronic Health Evaluation II
AST (SGOT)	Aspartate aminotransferase
AUC	Area under the curve
$AUC_{0-\infty}$	Area under the curve from 0 to infinity
AUC_{0-last}	Area under the concentration time curve from the time of the dose to the T_{last}
AUC_{0-tELF}	Area under the concentration time curve for the epithelial lining fluid
$AUC_{0-tPlasma}$	Area under the concentration time curve for plasma
β	Beta
BAL	Bronchoalveolar lavage
BLI	β-lactamase inhibitor
BLQ	Below limit of quantification
BUN	Blood urea nitrogen
CFR	Code of Federal Regulations
CPK	Creatine phosphokinase
cIAI	Complicated intra-abdominal infections
CL	Plasma clearance
C_{last}	Last quantifiable plasma concentration
CL_{CR}	Creatinine clearance
C_{max}	Maximum concentration
CRO	Contract research organization
CRP	C-reactive protein
Cr_{serum}	Serum creatinine
C_{tELF}	Concentration in the epithelial lining fluid
$C_{tPlasma}$	Concentration in the plasma
Cubist	Cubist Pharmaceuticals LLC, an indirect wholly-owned subsidiary of Merck Sharp & Dohme Corp.
cUTI	Complicated urinary tract infections
%CV	% Coefficient of variation

Abbreviation or Specialist Term	Explanation
CYP	Cytochrome
CYP450	Cytochrome P450
DCSI	Developmental Core Safety Information
EC	Ethics Committee
eCRF	Electronic case report form
EDC	Electronic Data Capture
ELF	Epithelial Lining Fluid
ESBL	Extended spectrum β-lactamase
ET	Early termination
FDA	Food and Drug Administration
GCP	Good Clinical Practice
GFR	Glomerular filtration rate
GLP	Good Laboratory Practice
h	Hour
ICH	International Conference on Harmonization
IP	Intraperitoneal
IRB	Institutional Review Board
IV	Intravenous
Kg	Kilogram
L	Liter
M	Meter
M1	Tazobactam metabolite
MedDRA	Medical Dictionary for Regulatory Activities
μg	Microgram
mg	Milligram
MIC	Minimum inhibitory concentration
Min	Minute
mITT	Modified Intent to Treat
mL	Milliliter
PBP	Penicillin-binding protein
PD	Pharmacodynamic
PICC	Peripherally inserted central catheter

Abbreviation or Specialist Term	Explanation
PK	Pharmacokinetic
PT	Preferred term
PTA	Probability of target attainment
q8h	Every 8 hours
RSI	Reference Safety Information
SAE	Serious adverse event
SD	Standard deviation
SI	International System of Units
SOC	System organ class
SUSAR	Suspected unexpected serious adverse reactions
$t_{½}$	Terminal elimination half-life
T_{last}	Time of last quantifiable plasma concentration
T>MIC	Time above the minimum inhibitory concentration (t>MIC)
T_{max}	Time of maximum concentration
$[urea]_{BAL}$	Concentration of urea in the bronchoalveolar lavage fluid
$[urea]_{ELF}$	Concentration of urea in the epithelial lining fluid
V_{BAL}	Volume of bronchoalveolar lavage fluid
V_{ELF}	Volume of epithelial lining fluid
V_{ss}	Volume of distribution at steady state
WBC	White blood count
WHO	World Health Organization

4. INTRODUCTION

4.1. Ceftolozane/tazobactam

Ceftolozane/tazobactam, a novel intravenous (IV) antibiotic with activity against *Pseudomonas aeruginosa*, including drug-resistant strains, and other common Gram-negative pathogens including extended spectrum β-lactamase (ESBL)-producing Enterobacteriaceae, is being developed for the treatment of complicated urinary tract infections (cUTIs), complicated intra-abdominal infections (cIAIs) and nosocomial pneumonia. Ceftolozane/tazobactam consists of ceftolozane, a unique anti-pseudomonal cephalosporin, and tazobactam, a well-established β-lactamase inhibitor. Cephalosporin antibiotics have been widely used in clinical practice because of their broad antibacterial spectrum, potent activity and excellent safety profile. Tazobactam in combination with piperacillin has also been widely used in clinical practice since 1994.

4.2. Non-Clinical Experience

Ceftolozane/tazobactam has been well characterized in a comprehensive series of in vitro and in vivo nonclinical studies that have defined key safety findings, including test-article related effects, and the reversibility of these changes.

Ceftolozane showed no potential to affect the functioning of the cardiovascular, respiratory, and central nervous systems across rats and dogs at clinically relevant exposures. The safety of tazobactam is supported by its long history of clinical use.

Ceftolozane and tazobactam, both alone and in combination, exhibited dose-proportional pharmacokinetics (PK) in rats and dogs with no gender differences and no change in systemic exposure with repeat dosing. Ceftolozane was distributed to tissues and exhibited low plasma protein binding, a predominantly renal route of elimination, and minimal metabolism following IV administration. Tazobactam exhibited low plasma protein binding and was hydrolyzed to a single major metabolite (M1) which lacks pharmacologic activity. No PK interactions were observed between ceftolozane and tazobactam when administered to rats or dogs in combination. In vitro, ceftolozane, tazobactam, and the M1 metabolite of tazobactam demonstrated low potential for drug-drug interactions involving cytochrome (CYP) enzymes and/or transporters at clinically relevant concentrations.

No target organ toxicities were identified for ceftolozane alone or in combination with tazobactam. Effects associated with ceftolozane and tazobactam treatment alone were limited to non-adverse changes in the kidney and liver, respectively. Ceftolozane-related effects in the kidney were limited to the microscopic presence of hyaline droplets in proximal renal tubule epithelium in the absence of toxicologically meaningful degeneration or necrosis with no effects on renal function noted as determined by the absence of biologically relevant changes in serum blood urea nitrogen (BUN), creatinine, inorganic phosphorus, urine volume, or presence of cellular/granular casts. Tazobactam-related effects in the liver of rats and dogs was observed as microscopic evidence of reversible glycogen accumulation in hepatocytes following twice daily repeat intraperitoneal (IP) or IV administration of tazobactam to rats and dogs. Importantly, no new effects or unexpected toxicities were observed when ceftolozane was combined with tazobactam. The weight of evidence suggests that ceftolozane alone and in combination with

tazobactam has a low potential for genotoxicity. Ceftolozane showed a potential for local tolerance effects in rodents, a low potential for antigenicity and reproductive effects, and no potential for phototoxicity, immunotoxicity, or hemolytic activity.

Overall, the nonclinical data package collected on ceftolozane/tazobactam support doses up to 3 g administered IV every 8 hours. Further information is available in the ceftolozane/tazobactam Investigator's Brochure.

4.3. Clinical Experience with Ceftolozane/tazobactam

Ceftolozane alone or ceftolozane/tazobactam has been evaluated in adults in 10 completed Phase 1 studies, a Phase 2 study in cUTI (ceftolozane alone), a Phase 2 study in cIAI, a Phase 3 study in cUTI and a Phase 3 study in cIAI.

4.3.1. Pharmacokinetics

The results from the adult Phase 1 and Phase 2 studies demonstrated ceftolozane/tazobactam PK characteristics are generally consistent with those of other renally excreted β-lactam antibiotics. Ceftolozane/tazobactam has linear PK across a wide range of doses (250 to 3000 mg for ceftolozane and 100 to 1500 mg for tazobactam), and its volume of distribution at steady state (V_{ss}), approximately 13 to 15 liters (L), is roughly equivalent to the extracellular fluid volume. The plasma protein binding is low, approximately 16 to 21% and 30% for ceftolozane and tazobactam, respectively. The terminal elimination half-life ($t_{½}$) for ceftolozane and tazobactam is 2 to 3 hours and approximately 1 hour, respectively, independent of dose, and with little or no accumulation after multiple dosing every 8 hours. Ceftolozane/tazobactam is primarily cleared from the systemic circulation into the urine by glomerular filtration, but tazobactam is also partly cleared by tubular secretion. About 20% of the tazobactam dose is converted to M1 via hydrolysis of the β-lactam ring while >95% of administered ceftolozane dose is eliminated as unchanged parent drug, suggesting minimal potential for cytochrome P450 (CYP450) mediated metabolism. Co-administration of ceftolozane/tazobactam given as 1-hour IV infusions in a fixed 2:1 ratio of ceftolozane to tazobactam does not change the pharmacokinetic profiles of either drug, nor of the tazobactam M1 metabolite, indicating no drug-drug interaction between ceftolozane and tazobactam.

Ceftolozane/tazobactam is primarily renally eliminated therefore creatinine clearance (CL_{CR}) is the primary factor that influences and predicts exposure; no other clinically meaningful intrinsic factors affecting the exposure have been identified. A drug-drug interaction study suggests a low potential for any CYP450 or transporter-mediated PK interaction, and there is a low potential for PK interaction with other drugs eliminated primarily by glomerular filtration, such as tobramycin and vancomycin.

Ceftolozane/tazobactam pharmacokinetics has been studied in healthy subjects and subjects with differing levels of renal impairment in single- and repeat-dose studies. No clinically relevant differences in exposure were observed in subjects with mild renal impairment (CL_{CR} >50 to 89 mL/min), whereas exposures increased approximately 2- to 2.5-fold and 3- to 5-fold in subjects with moderate (CL_{CR} 30 to 50 mL/min) and severe (CL_{CR} 15 to 29 mL/min) renal impairment, respectively. Based on these results and probability of target attainment, subjects with mild renal impairment do not require a dosage adjustment; however, a 2- and 4-fold dose reduction is recommended in subjects with moderate and severe renal impairment, respectively.
A Phase 1 intrapulmonary PK study (CXA-ELF-10-03) evaluated concentrations within the

epithelial lining fluid (ELF) of 50 healthy subjects following administration of intravenous ceftolozane/tazobactam (1.5 g) or piperacillin/tazobactam (4.5 g). Plasma and ELF concentrations increased rapidly following the start of infusion and reached maximum concentrations at the end of infusion. The penetration ratio of the ceftolozane component of ceftolozane/tazobactam in the ELF was 48%, while that of the piperacillin component of piperacillin/tazobactam was 26%. The penetration ratio of tazobactam when dosed as ceftolozane/tazobactam and piperacillin/tazobactam was 44% and 54%, respectively.

4.3.2. Safety

In adult patients, 2 Phase 1 and 1 Phase 2 clinical studies have been completed with ceftolozane alone, and 8 Phase 1, 1 Phase 2, and 2 Phase 3 clinical studies in 2 indications have been completed with ceftolozane/tazobactam. A total of 145 subjects in these clinical studies received ceftolozane alone, and 1294 received ceftolozane/tazobactam.

Phase 1 studies suggested ceftolozane/tazobactam was safe and well tolerated. The most common adverse events (AEs) in Phase 1 studies were mainly gastrointestinal in origin (nausea, vomiting, constipation and diarrhea), as well as headache and infusion site reactions. Multiple dose studies (CXA-101-01, CXA-201-01, and CXA-MD-11-07) demonstrated that doses of ceftolozane/tazobactam up to 9 g daily (3 g every 8 hours [q8h]) were well tolerated in adults for durations of up to 10 days. A thorough QT study (CXA-QT-10-02) demonstrated that therapeutic and 3-fold supra-therapeutic doses of ceftolozane/tazobactam did not increase QTc, QTcF and QTcB intervals, and no findings indicated an effect of ceftolozane/tazobactam on cardiac repolarization. Only one serious adverse event (SAE) was reported in the 9 Phase 1 studies for ceftolozane or ceftolozane/tazobactam; one patient in study CXA-REN-11-01had an unrelated SAE of thrombosis of arteriovenous fistula reported on study day 44 that required hospitalization for heparinization and catheter replacement. Adverse events leading to discontinuation of ceftolozane/tazobactam in the Phase 1 studies were rare. In Phase 2 studies of ceftolozane in cUTI (CXA-101-03) and ceftolozane/tazobactam in cIAI (CXA-IAI-10-01), the most common AEs across both studies were pyrexia, nausea, constipation, sleep disorder, anemia, headache, vomiting, diarrhea and insomnia. In study CXA 101-03, there was an imbalance in the incidence of hyperglycemia severity grade shifts which was neither associated with ongoing abnormalities in serum glucose nor reported as adverse events. In study CXA-IAI-10-01, there was an imbalance in the incidence of hemoglobin severity grade shifts in the ceftolozane/tazobactam group; however the decreases in hemoglobin appeared to be related to complicated surgical procedures in high-risk patients or the patient’s underlying condition. Collectively, 3 patients experienced a shift from negative direct Coombs’ test at baseline to positive at the test of cure (TOC) visit, but none were associated with a report of hemolytic anemia.

In the integrated Phase 3 studies of ceftolozane/tazobactam in cUTI and cIAI (CXA-cUTI-10-04, CXA-cUTI-10-05, CXA-cIAI-10-08, and CXA-cIAI-10-09), 1015 adult patients received treatment with ceftolozane/tazobactam. The most common AEs from these trials were nausea, headache, diarrhea, pyrexia, constipation, hypertension, insomnia, and vomiting. Overall SAE rates were 5.3% and 5.2% in the ceftolozane/tazobactam and comparator arms, respectively. Drug discontinuation rates due to AEs were similar, with 2% of patients who discontinued ceftolozane/tazobactam and 1.9% of patients who discontinued comparator drug. Drug related SAEs with ceftolozane/tazobactam were limited to only 2 cases of *Clostridium difficile* colitis

compared to 1 case with comparator drug. In cUTI, there was 1 death in the ceftolozane/tazobactam treatment arm and none in the comparator arm. In cIAI, there were 11 deaths in the ceftolozane/tazobactam arm and 8 deaths in the comparator arm. All of deaths reported in these Phase 3 studies were deemed unrelated to study therapy by the Investigator.

An open-label Phase 3 study in nosocomial pneumonia was terminated after enrollment of 4 patients. Two patients experienced SAEs, 1 in each arm, and resulted in death in the ceftolozane/tazobactam treatment arm. Neither event was deemed to be related to study drug. A new Phase 3 study in nosocomial pneumonia is ongoing.

4.4. Rationale

4.4.1. Study Rationale

Nosocomial pneumonia is one of the most common hospital-acquired infections, can be life-threatening, and often occurs in patients with mechanical ventilation. Gram-negative bacteria are commonly implicated in cases of nosocomial pneumonia, most notably *Pseudomonas aeruginosa* (including multi-drug resistant strains), Enterobacteriaceae (including ESBL-producing strains), and *Acinetobacter baumannii* [1, 2]. The choice of appropriate empiric antibiotic therapy for nosocomial pneumonia is typically based on local resistance epidemiology data. The dose used will often have been based on the PK and pharmacodynamics (PD) of the antibiotic therapy, including concentrations at the site of infection. Despite limited data correlating clinical outcome and concentration at the site of infection in nosocomial pneumonia where target pathogens are located (ie, ELF), achieving clinically significant concentrations in the ELF remains important in dose selection [3].

A study in healthy volunteers demonstrated that ceftolozane/tazobactam penetrates well into the intrapulmonary space [4]. Extrapolation of these results to patients with infected lungs is challenging due to the multiple factors (e.g., capillary leak, fluid loading, and altered renal function) that may distort the normal pharmacokinetic profile. Due to the potential for local inflammatory response to alter study drug penetration into the lung, plasma and BAL samples will be evaluated for ceftolozane/tazobactam concentrations. In addition, modifications of albumin concentration in ELF are associated with the presence of inflammatory processes in the lower respiratory tract, and C-reactive protein (CRP) is a well-known biochemical marker of inflammation [5, 6]. Therefore, CRP and albumin will also be assessed to identify any potential association between the patients' inflammatory response and ceftolozane/tazobactam penetration into the ELF. Collectively, data generated will help understand how nosocomial pneumonia pathophysiology affects the PK of ceftolozane/tazobactam.

Augmented renal clearance (ARC) has been reported in critically ill patients, can result in sub-therapeutic plasma concentrations of renally excreted antibiotics, and has been associated with a higher incidence of therapeutic failure and worse clinical outcomes [7, 8]. Ceftolozane/tazobactam is primarily renally eliminated, therefore maximizing time above the minimum inhibitory concentration (T>MIC) may prove challenging in this patient population. The prevalence of ARC has not been consistently defined in the literature, varying from CL_{CR} > 120 to 160 mL/min [9, 10]. In adult Phase 2 and 3 cUTI and cIAI studies with ceftolozane/tazobactam, approximately 2-6% of patients exhibited CL_{CR} greater than 180 mL/min. To supplement this existing PK database for ceftolozane/tazobactam, this study

will characterize the plasma concentrations of ceftolozane/tazobactam in critically ill patients with a $CL_{CR} \geq 180$ mL/min. This information can be used to optimize the dosage regimen of ceftolozane/tazobactam in those with augmented renal clearance.

4.4.2. Dose Rationale

The 3 g dose of ceftolozane/tazobactam for pneumonia was selected based on (a) results from a Phase 1 ELF PK study conducted in healthy volunteers showing that the ELF/plasma area under the curve (AUC) ratio is approximately 0.5 [4], and (b) PK/PD modeling using concentrations from the ELF and plasma of 25 healthy subjects and a distribution of pathogens, selected from patients hospitalized with pneumonia, from the 2012 Program to Assess Ceftolozane/Tazobactam Susceptibility (PACTS) surveillance data in the United States and European Union. The target for bactericidal activity was 32.2% fT>MIC (i.e., 1-log kill), and a 3 g dose of ceftolozane/tazobactam given as a 60-minute IV infusion every 8 hours achieved approximately 99% probability of target attainment (PTA) against common respiratory pathogens with an MIC up to 8 µg/mL in ELF. The 3 g dose was selected for this study to further evaluate the clinical dose to be studied in patients with pneumonia.

5. TRIAL OBJECTIVES AND PURPOSE

5.1. Primary Objective

The primary objective of this study is to characterize the plasma PK and intrapulmonary penetration, as measured by concentrations in the ELF, of ceftolozane/tazobactam in mechanically ventilated patients concurrently receiving standard antibiotic therapy for proven or suspected pneumonia.

5.2. Secondary Objectives

- Characterize the plasma PK of ceftolozane/tazobactam after a single dose in critically ill patients with a $CL_{CR} \geq 180$ mL/min (as calculated by the Cockcroft-Gault equation).
- Assess the safety and tolerability of ceftolozane/tazobactam.

5.3. Other Objectives

- To characterize the effect of inflammation on the plasma and intrapulmonary PK of ceftolozane/tazobactam in pneumonia using inflammatory markers such as CRP and albumin from plasma and BAL samples. Furthermore, cytology counts will be performed on BAL samples to improve accuracy of PK sample analysis.
- To explore the correlation between baseline measured CL_{CR} (from the 8-hour urine collection) and baseline estimated CL_{CR} (as calculated from the Cockcroft-Gault equation).

6. INVESTIGATIONAL PLAN

6.1. Overall Study Design

This is a Phase 1, prospective, multi-center, open-label study of ceftolozane/tazobactam being conducted in 2 groups of patients:

- **<u>Group 1:</u>** Approximately 25 ventilated patients with proven or suspected pneumonia receiving concurrent standard antibiotic therapy. Within Group 1, efforts will be made to enroll approximately 5 patients with a $CL_{CR} \geq 150$ mL/min (as calculated by the Cockcroft-Gault equation);
- **<u>Group 2:</u>** 8-10 critically ill patients with $CL_{CR} \geq 180$ mL/min (as calculated by the Cockcroft-Gault equation).

Patients in Group 1 will receive ceftolozane/tazobactam every 8 hours for a total of 4-6 doses, with the exception of patients with severe renal impairment (CL_{CR} 15 – 29 mL/min) at baseline who must receive a total of 6 doses. All patients in Group 1 will have blood and bronchoalveolar lavage (BAL) samples collected for PK analysis. Each patient in Group 1 will have 12 PK blood samples collected and will undergo only one BAL procedure for this study; the timing of each BAL sample will be assigned and will be collected after the last dose of study drug. There will be approximately 5 patients per BAL time point in Group 1. An interim PK analysis will be performed after 3 BAL samples at each scheduled sampling time point are available. Enrollment in Group 1 may stop early if the estimated overall inter-individual variability is not higher than or the estimated minimum penetration ratio is not lower than that observed in the previous ELF study in healthy volunteers. Enrollment in Group 1 may continue beyond 25 patients if the penetration ratio cannot be reliably estimated.

Patients in Group 2 will receive a single dose of ceftolozane/tazobactam (3 g IV once) and will have blood samples collected for plasma PK analysis. Patients in Group 2 will have 6 PK blood samples collected but will not undergo a BAL procedure.

Baseline (screening) procedures, including medical and surgical history, demographics, vital signs, serum pregnancy test (as applicable), and clinical laboratory tests (hematology, chemistry, coagulation) should be performed as close as possible to the start of study drug administration and at most within 24 hours prior to dosing.

In all patients who are catheterized, 8- hour urine collections will be obtained for direct measurement of CL_{CR}. Exploratory analysis will be performed to compare baseline measured CL_{CR} with baseline estimated CL_{CR} (as calculated from the Cockcroft-Gault equation).

Group 1 will require a minimum commitment of 3 days and a maximum of 5 days. Patients in Group 2 will require a commitment of 2 days. Assessment of adverse events and concomitant medications and procedures will occur during the study until the follow-up visit, 24-48 hours after the last dose of study drug.

6.1.1. Dosing and the Interim PK Assessment

The doses of ceftolozane/tazobactam to be administered to patients in Group 1 are summarized in Table 1: Ceftolozane/tazobactam Dosing in Group 1.

Table 1: Ceftolozane/tazobactam Dosing in Group 1

Renal Function	Dose	Infusion Frequency	Duration of Infusion
CL_{CR} > 50 mL/min	3 g IV	q8h (± 1 hours)	60 ± 10 min infusion
CL_{CR} 30 – 50 mL/min	1.5 g IV	q8h (± 1 hours)	60 ± 10 min infusion
CL_{CR} 15 – 29 mL/min	750 mg IV	q8h (± 1 hours)	60 ± 10 min infusion

CL_{CR} = Creatinine clearance; IV = Intravenous; q8h = Every 8 hours

The dose of ceftolozane/tazobactam should be adjusted accordingly based on post-baseline assessments of creatinine clearance (i.e. calculated or measured).

All patients in Group 2 will receive a single 3 g IV dose of ceftolozane tazobactam as a 60 ± 10 minute infusion.

In Group 1, an interim population PK analysis will be conducted after 3 BAL samples at each scheduled sampling time point are available. Further information is provided in Section 12.2.4.

6.2. Study Visits

Baseline assessments will occur within 24 hours of study drug administration. Assessment of AEs and concomitant medications and procedures will occur during the study up to 24-48 hours after the last dose of study drug at follow-up.

6.3. Study Procedures

The schedules of assessments for the study are summarized in Table 2 and Table 3 .

6.3.1. Clinical Assessments

- Medical and surgical history from the past 3 years
- Prior and concomitant medications (excluding supplements) given within 7 days prior to the first dose of study drug through the last study evaluation (follow-up)
- Vital sign assessment including blood pressure, heart rate, respiratory rate and temperature (oral, rectal, tympanic, or core)
- Weight and height
- Estimate the patient's CL_{CR} using the patient's serum creatinine (Cr_{serum}) value, actual body weight, and the Cockcroft-Gault formula below:

$$CL_{CR}\ (\text{mL/min}) = \frac{(140 - \text{Age[years]}) \times \text{Weight[kg]} \times (0.85 \text{ if female})}{72 \times Cr_{serum}\ [\text{mg/dL}]}$$

Note: 1 mg/dL= 88.4 μmol/L

- Assessment of AEs, as detailed in Section 11

6.3.2. Laboratory Assessments

- Blood samples for hematology (hemoglobin, hematocrit, white blood cell [WBC] count with differential, and platelet count), serum chemistry (alkaline phosphatase, albumin, blood urea nitrogen [BUN], creatinine, non-fasting serum glucose, potassium, bicarbonate, chloride, magnesium, creatine phosphokinase (CPK), alanine aminotransferase [ALT], aspartate aminotransferase [AST], sodium, total bilirubin, total protein) and coagulation (prothrombin time) evaluation
- Serum pregnancy test for female patients who are of child bearing potential
- In all patients who are catheterized, an 8-hour urine collection will be obtained. Collection must be started prior to the first dose, but may end after the first dose. Calculate measured CL_{CR}:

$$CL_{CR}\ (\text{mL/min}) = \frac{\text{urine creatinine [mg/dL]} \times \text{total urine volume over 8 hour period [mL]}}{Cr_{serum}\ [\text{mg/dL}] \times 8 \text{ hours} \times 60 \text{ minutes}}$$

6.3.3. Pharmacokinetic and Inflammatory Markers Sampling

6.3.3.1. Blood Sampling

- Collect blood samples (5 mL per sample) at the following time points as detailed in Section 10.1:
  - **For Group 1:** 0 (pre-dose), 1, 2, 4, 6, 8 hours after the start of the first and final (4th, 5th or 6th) infusions. Patients with severe renal impairment (CL_{CR} 15 – 29 mL/min) at baseline must receive a total of 6 doses. The time point windows are as follows (all time points are in hours post start of study drug administration): 1 (+ 5 minutes), 2 (± 10 minutes), 4 (± 10 minutes), 6 (± 20 minutes), 8 (± 20 minutes).

    Note: Blood samples collected at the time of BAL sampling will also be tested for inflammatory markers
  - **For Group 2:** 0 (pre-dose), 1, 2, 4, 6, and 8 hours after the start of the infusion. The time point windows are as follows (all time points are in hours post start of study drug administration): 1 (+ 5 minutes), 2 (± 10 minutes), 4 (± 10 minutes), 6 (± 20 minutes), 8 (± 20 minutes).

NOTE: The 1 hour time point is equivalent to the end of the study drug infusion. PK blood samples should be taken **just after** the total dose is administered and the infusion line is flushed (as per standard of care).

6.3.3.2. BAL Sampling (Group 1)

- As detailed in Section 10.2, patients will undergo one bronchoscopy and BAL at 1, 2, 4, 6, or 8 hours after the start of the final (4th, 5th, or 6th) infusion. Patients with severe renal impairment (CL_{CR} 15 – 29 mL/min) at baseline must receive a total of 6 doses. The time point windows are as follows: (all time points are in hours post start of study drug administration): 1 (+ 30 minutes), 2 (± 30 minutes), 4 (± 30 minutes), 6 (± 30 minutes), 8 (± 30 minutes).

  NOTE: The 1 hour time point is equivalent to the end of the study drug infusion. BAL Sampling should be performed **after** the total dose is administered and the infusion line is flushed (as per standard of care).

- In the event that a patient in Group 1 undergoes a BAL procedure as part of standard of care (at any time point post-dose during the study), an additional sample may be collected for PK analysis.

6.4. Number of Patients

Approximately 25 patients are planned to be enrolled into Group 1 and 8-10 patients are planned to be enrolled into Group 2. Within Group 1, efforts will be made to enroll approximately 5 patients with a $CL_{CR} \geq 150$ mL/min.

6.5. Treatment Assignment

Patients in Group 1 will receive ceftolozane/tazobactam every 8 hours for a total of 4-6 doses, with the exception of patients with severe renal impairment (CL_{CR} 15 – 29 mL/min) at baseline

who must receive a total of 6 doses (Table 1). Patients in Group 2 will receive a single dose of ceftolozane/tazobactam (3 g IV).

6.6. Criteria for Study Termination

Cubist reserves the right to terminate the study at any time. Reasons for termination at any particular site includes, but are not limited to, the following: safety or AE finding that poses a potentially significant risk to the patients, site non-compliance with protocol and regulatory requirements, institutional review board (IRB)/ethics committee (EC) or the Investigator's decision to terminate or suspend the study at the investigative site.

Table 2: Schedule of Assessments – Group 1

Procedure	Baseline[a]	Ceftolozane/tazobactam Infusion Number				Follow-up[b]
		1	2	3	Final (4, 5, or 6)	
Informed Consent	X					
Medical/Surgical and Medication History	X					
Demographics	X					
Inclusion/Exclusion	X					
Assign BAL Sampling Time Point [c]	X					
Vital Signs	X[d]	X[e]			X[e]	X
Serum Pregnancy Test [f]	X					
Calculated Creatinine Clearance	X	X[g]			X[g]	X
Chemistry/Hematology/Coagulation	X					X
8-hour Urine Collection	X[h]					
Concomitant Medications and Procedures [i]	X	X	X	X	X	X
Adverse Event Assessment		X	X	X	X	X
Blood Sampling [j]		X			X	
BAL Sampling [k]					X	

a. Completed within 24 hours prior to dosing
b. 24-48 hours after the last dose of study drug
c. Refer to the BAL Assignment Manual
d. Includes height and weight
e. Vital sign assessments (blood pressure, heart rate, respiratory rate, and temperature [oral, rectal, tympanic or core]) will be conducted within 2 hours after the first and fourth ceftolozane/tazobactam infusions
f. For female patients of child-bearing potential
g. While on study drug, CL_{CR} should be calculated at a consistent time each day
h. In patients who are catheterized, baseline collection must be started prior to the first dose, but may end after the first dose
i. Collection of concomitant medications and procedures for 7 days prior to first dose of study drug through follow-up
j. Blood samples will be collected at the following time points: 0 h (pre-dose), 1 h (+ 5 min), 2 h (± 10 min), 4 h (± 10 min), 6 h (± 20 min), and 8 h (± 20 min) after the start of infusion. The 1 h sample should be obtained just after dose administration is completed.
k. 1 BAL time point per patient will be assigned at 1 h (+ 30min), 2 h (± 30min), 4 h (± 30min), 6 h (± 30min), or 8 h (± 30min) post start of final (4th, 5th, or 6th) infusion

Table 3: Schedule of Assessments – Group 2

**Procedure**	**Baseline**[a]	**Day of Ceftolozane/tazobactam Infusion**	**Follow-Up**[b]
Informed Consent	X		
Medical/Surgical and Medication History	X		
Demographics	X		
Inclusion/Exclusion	X		
Calculated APACHE II Score [c]	X		
Vital Signs	X[d]	X[e]	X
Serum Pregnancy Test [f]	X		
Calculated Creatinine Clearance	X		X
Chemistry/Hematology/Coagulation	X		X
8-hour Urine Collection	X[g]		
Concomitant Medications and Procedures [h]	X	X	X
Adverse Event Assessment		X	X
Blood Sampling [i]		X	

a. Completed within 24 hours prior to dosing.
b. 24-48 hours after the dose of study drug
c. Acute Physiology and Chronic Health Evaluation II Score (Section 21.1)
d. Includes height and weight
e. Vital sign assessments (blood pressure, heart rate, respiratory rate, and temperature [oral, rectal, tympanic or core]) will be conducted within 2 hours after the ceftolozane/tazobactam infusion
f. For female patients of child-bearing potential
g. In patients who are catheterized, baseline collection must be started prior to the first dose, but may end after the first dose
h. Collection of concomitant medications and procedures for 7 days prior to study drug administration through follow-up
i. Pharmacokinetic blood samples will be collected at the following time points: 0 h (pre-dose), 1 h (+ 5 min), 2 h (± 10 min), 4 h (± 10 min), 6 h (± 20 min), and 8 h (± 20 min) after the start of infusion. The 1 h sample should be obtained just after dose administration is completed.

7. SELECTION AND WITHDRAWAL OF PATIENTS

7.1. Patient Inclusion Criteria

1. Provide written informed consent prior to any study-related procedure not part of normal medical care. If the patient is unable to do so, local country laws and institution specific guidelines and requirements in place for obtaining informed consent should be met. A legally acceptable representative may provide consent, provided this is approved by local country and institution specific guidelines. If a patient is able to provide consent while still in the study and per the Investigator's judgment the patient is able to read, assess, understand and make his/her own decision to participate in the trial, the patient must agree to continue study participation and the patient may be re-consented, if required by local country and institution specific guidelines;
2. If female, patient must not be pregnant or nursing, and is either:
    a. Not of childbearing potential, defined as postmenopausal for at least 1 year or surgically sterile due to bilateral tubal ligation, bilateral oophorectomy, or hysterectomy; or
    b. Of childbearing potential and:
        - Is practicing an effective method of contraception (e.g., oral/parenteral contraceptives or a barrier method) and for at least 1 month prior to baseline assessments, or
        - Has a vasectomized partner, or
        - Is currently abstinent from sexual intercourse.

    Patients must be willing to practice the chosen contraceptive method or remain abstinent during the conduct of the study and for at least 30 days after last dose of study medication.
3. Non-vasectomized males are required to practice effective birth control methods (e.g., abstinence, use of a condom or use of other barrier device) during the conduct of the study and for at least 30 days after last dose of study medication;
4. Patients in Group 1 must meet the following criteria:
    a. Male or female patients age 18 years or older;
    b. Intubated and on mechanical ventilation for at least 24 hours prior to time of enrollment (includes patients with tracheostomy who are mechanically ventilated) and anticipated to be on mechanical ventilation for at least 8 hours following the final dose of study drug;
    c. Proven or suspected bacterial pneumonia, as confirmed by the presence of **at least one** of the following clinical signs and symptoms within the past 48 hours:
        - Documented fever (oral, rectal, tympanic, or core temperature > 38.5°C)
        - Hypothermia (oral, rectal, tympanic, or core temperature < 35.0°C)
        - An elevated white blood cell (WBC) count ≥12,000 cells/mm3
        - Radiological findings suggestive of bacterial pneumonia
    d. Receiving antibiotic therapy for proven or suspected bacterial pneumonia at the time of enrollment and expected to continue on antibiotic therapy while in the study

5. Patients in Group 2 must meet the following criteria:
    a. Male or female aged 18 – 54 years;
    b. APACHE II score between 12 and 35, inclusive (Section 21.1);
    c. Creatinine clearance (CL_{CR}) ≥180 mL/min (as calculated by the Cockcroft-Gault equation using actual body weight) within 24 hours of dosing;
    d. Documented infection or presumed infection as confirmed by the presence of **at least** one of the following criteria within the past 72 hours:
        - Documented fever (oral, rectal, tympanic, or core temperature > 38.5°C)
        - Hypothermia (oral, rectal, tympanic or core temperature < 35.0°C)
        - An elevated white blood cell (WBC) count ≥12,000 cells/mm3

7.2. Patient Exclusion Criteria

1. Has a documented history of any moderate or severe hypersensitivity or allergic reaction to any β-lactam antibacterial (a history of a mild rash followed by uneventful re-exposure is not a contraindication to enrollment);
2. Hemoglobin < 7 gm/dL at baseline;
3. Prior (within 24 hours of first dose of study drug) or concomitant receipt of piperacillin/tazobactam, probenecid, or ceftolozane/tazobactam (non-study use);
4. Any rapidly-progressing disease or immediately life-threatening illness (defined as imminent death within 48 hours in the opinion of the Investigator);
5. Any condition or circumstance that, in the opinion of the Investigator, would compromise the safety of the patient or the quality of study data;
6. Planned or prior participation in any interventional drug study within the last 30 days;
7. Patients in Group 1 must not meet any of the following criteria:
    a. Receipt of effective systemic antibiotic therapy for the treatment of proven or suspected bacterial pneumonia for more than 72 hours prior to start of the first dose of study drug

        Note: If signs and/or symptoms of bacterial pneumonia have not improved despite >72 hours of the antibacterial regimen, a patient is still eligible. This will require discussion with and approval from a medical monitor
    b. Any of the following diagnoses or conditions that may interfere with the PK assessment/interpretation:
        - Cystic fibrosis, acute exacerbation of chronic bronchitis or obstructive airway disease, chronic severe respiratory disease , or active pulmonary tuberculosis,
        - Full thickness burns (greater than 15% of total body surface area),
        - Lung transplant recipient or donor,
        - Any condition or situation where bronchoscopy is not advisable;
    c. End-stage renal disease defined as a CL_{CR} < 15 mL/min (as calculated by the Cockcroft-Gault equation using actual body weight), OR requirement for continuous renal replacement therapy or hemodialysis.

7.3. Patient Withdrawal Criteria

Patients may withdraw from the study at any time without prejudicing their medical care and are not obliged to state their reasons. In addition, patients may be withdrawn, if deemed necessary for their health and safety, by the Investigator or Cubist. Patients that discontinue for any reason should be asked to provide the reason(s) for discontinuation and assessments should be performed as outlined in the Schedule of Assessments for Follow-up (Table 2 and Table 3) Reasons for withdrawal from the study will be summarized. Any discontinuations must be fully documented in the electronic case report form (eCRF).

7.4. Replacement of Patients

Patients who are withdrawn from the study before receiving study drug (or before undergoing a BAL sample in Group 1) will be replaced in order to enroll approximately 25 patients in Group 1 and 8-10 patients in Group 2. Patient identification numbers will not be reused.

8. TREATMENT OF PATIENTS

8.1. Description of Study Drug

Study medication will be supplied by Cubist for use in this protocol and is for investigational use only. Please refer to the current Investigator's Brochure for additional information.

**Product Name:**	ceftolozane/tazobactam
**Dosage Form:**	IV infusion over 60 minutes
**Route of Administration:**	Intravenous
**Physical Description:**	When a 1.5 g dose of ceftolozane/tazobactam lyophilized powder is reconstituted and diluted into an IV bag for use, ceftolozane/tazobactam contains 1 g of active ceftolozane and tazobactam sodium at a quantity equivalent of 500 mg of tazobactam free acid. Inactive ingredients include sodium chloride as a stabilizer and L-arginine and citric acid for pH adjustment.
**Manufacturer:**	Cubist Pharmaceuticals LLC, an indirect wholly-owned subsidiary of Merck Sharp & Dohme Corp.

8.2. Concomitant Medications

Any procedure performed and any concomitant medication (excluding supplements) given within 7 days prior to the first dose of study drug through the last study evaluation (Follow-up) must be recorded in the appropriate section of the eCRF.

The use of the following medications are prohibited: piperacillin/tazobactam, probenecid, or ceftolozane/tazobactam (non-study use).

8.3. Patient Identification and Numbering

At time of consent, patients will receive a unique number specific to the site. The full Patient Identification Number will consist of a three-digit site number and a four-digit patient number separated by a hyphen (ex: XXX-XXXX).

8.4. BAL Assignment (Group 1)

In conjunction with assignment of the patient number, the investigator or designee will also obtain assignment of BAL sampling time point. Patients will be assigned one BAL time point at 1 h (+ 30 min), 2 h (± 30 min), 4 h (± 30 min), 6 h (± 30 min), or 8 h (± 30 min) post start of the final (4th, 5th, or 6th) infusion. Patients will be blocked into groups of 5 and will be assigned sampling time points in a manner that ensures each time point has been assigned before proceeding to the next block.

9. STUDY DRUG MATERIALS AND MANAGEMENT

9.1. Study Drug

Study medication will be supplied by Cubist for use in this study and is for investigational use only.

9.2. Study Drug Packaging, Labeling and Storage

Labeling and packaging of study medication will meet applicable regulatory requirements. Study medication must be stored in a secure, limited access area, and may be dispensed only by specifically authorized personnel. Refer to the Pharmacy Manual for further details on packaging, labeling, and storage.

9.3. Study Drug Preparation

Refer to the Pharmacy Manual for step-by-step directions for ceftolozane/tazobactam preparation.

9.4. Administration

Group 1 patients will receive IV ceftolozane/tazobactam as 60 ± 10 minute infusions every 8 (±1) hours for a total of 4-6 doses. Patients with severe renal impairment (CL_{CR} 15 – 29 mL/min) at baseline must receive a total of 6 doses (Table 1).

Group 2 patients will receive a single dose of 3 g IV ceftolozane/tazobactam as a 60 ± 10 minute infusion.

9.5. Study Drug Accountability

The site will maintain accurate inventory and dispensing records. Unused study medication must not be discarded nor used for any purpose other than the present study. The study monitor will review the drug accountability forms prior to arranging for return or destruction of all study medication. Refer to the Pharmacy Manual for further details on study drug accountability.

10. PHARMACOKINETIC ASSESSMENTS

10.1. Blood Sample Collection

Blood samples (5 mL per sample) for plasma PK determination of ceftolozane and tazobactam will be collected in all patients. Sodium heparin will be used as the anticoagulant. Samples should be taken following flushing of the line (as per standard of care).

For patients in Group 1, samples will be collected at the following time points: 0 (pre-dose) and at 1, 2, 4, 6, and 8 hours after the start of the first and final (4th, 5th or 6th) infusions. Patients in this group will also have a BAL performed at an assigned time point; blood samples corresponding with this time point will be split for PK and Inflammatory Marker (IM) analyses. For patients in Group 2, samples will be collected at the following time points: 0 (pre-dose) and at 1, 2, 4, 6, and 8 hours after the start of the infusion.

The time point windows are as follows: (all time points are in hours post start of study drug administration): 1 (+ 5 minutes), 2 (± 10 minutes), 4 (± 10 minutes), 6 (± 20 minutes), 8 (± 20 minutes). The exact time of sample collection will be recorded.

> **NOTE:** The 1 hour time point is equivalent to the end of the study drug infusion. PK blood samples should be taken **just after** the total dose is administered and the infusion line is flushed (as per standard of care).

For the **0, 2, 4, 6, and 8** hour time points, if reasonable effort to gain a second line is unsuccessful, a single IV line (or comparable means of venous access) is acceptable for both drug administration and sample collection.

Specifically for the 1 hour time point, if access from either a second line or an individual peripheral phlebotomy is both not possible, a single IV line for both drug administration and sample collection may **only** be used after waiting 2 minutes from the end of infusion. The sample should still be collected within 5 minutes of the end of infusion.

The method of sampling for PK blood draws will occur at the discretion of the Investigator (e.g., peripherally inserted central catheter [PICC] line, indwelling catheter access, individual peripheral phlebotomies). PK samples can be obtained from a peri-operatively placed arterial line. In circumstances in which the blood draw and study medication infusion must occur in the same limb, the blood must be drawn more distally in the vein (3-5 inches) than the infusion site. The route of sampling must be indicated on the eCRF.

10.2. BAL Specimen Collection (Group 1)

Patients will undergo one bronchoscopy and BAL at 1, 2, 4, 6, or 8 hours after the start of the final (4th, 5th, or 6th) infusion of study drug. This BAL time point is assigned.

The time point windows are as follows: (all time points are in hours post start of study drug administration): 1 (+ 30 minutes), 2 (± 30 minutes), 4 (± 30 minutes), 6 (± 30 minutes), 8 (± 30 minutes). In patients who undergo a BAL procedure as part of standard of care (at any time point post-dose during the study), an additional sample may be collected for PK analysis. The exact time of sample collection will be recorded.

NOTE: The 1 hour time point is equivalent to the end of the study drug infusion. BAL Sampling should be performed **after** the total dose is administered and the infusion line is flushed (as per standard of care).

A fiberoptic bronchoscope will be inserted and, after routine inspection of the respiratory tract and aspiration of tracheobronchial secretions, wedged into the lung segment identified radiologically as that containing the infection.

Following proper positioning of the bronchoscope, one initial 50 mL aliquot, followed by three 20 mL aliquots of sterile 0.9% normal saline will be instilled into the lung segment identified radiologically as that containing pneumonia, and each specimen will be immediately aspirated and placed in ice. The aspirate from the first 50 mL instillation will be collected separately and discarded. The aspirates recovered from the second, third, and fourth instillations will be pooled. The volume of the pooled BAL will be measured and recorded.

10.3. Sample Handling and Processing

Human plasma and BAL samples collected for analysis of ceftolozane and tazobactam will be sent to MicroConstants (San Diego, CA, USA) for analysis. At the assigned BAL time point, samples will also be analyzed for Inflammatory Markers, and sent to Frontage Lab (Exton, PA, USA). Please reference the PK Sample Collection, Handling, and Shipping Instructions Manual (Sample Handling Guidelines) for details.

10.3.1. Blood Sample Handling and Processing

Uniquely-labeled polypropylene cryotubes will be prepared ahead of the blood sample collection and pre-chilled by refrigeration. Collect at least 5.0 mL of blood using a sodium heparin vacutainer tube. The blood tubes will be mixed by inverting 5 times and kept on ice after collection. The blood will be centrifuged within 30 minutes of collection (approximately 3000 rpm [approximately 2056 x g] for 15 to 20 minutes to achieve a clear plasma layer over the red cells) at approximately 4°C.

Samples will be aliquotted into cryotubes as described in detail in the Sample Handling Guidelines. As soon as the samples are placed in the cryotubes, they must be immediately frozen and stored at -80°C ± 10°C at all times in an upright position.

These samples are to be shipped according to the instructions in the Sample Handling Guidelines.

10.3.2. BAL Sample Handling and Processing

Following collection and pooling, the BAL samples will be centrifuged for 5 minutes at 400 G force at approximately 4°C. All BAL samples must be kept on ice, and frozen at -80°C ± 10°C within 17 hours of collection.

Ceftolozane/tazobactam

Immediately after centrifugation, BAL samples will be aliquotted into 15 mL Amber Specimen Bottles (labeled A, B, and C) according to the instructions in the Sample Handling Guidelines.

Inflammatory Markers

Immediately after centrifugation, BAL samples will be aliquotted into 15 mL Amber Specimen Bottles (labeled D, E, and F) according to the instructions in the Sample Handling Guidelines.

All BAL samples must be stored frozen at -80°C ± 10°C at all times and shipped according to the instructions in the Sample Handling Guidelines.

Calculation of ELF volume will be determined by the urea dilution method, using urea as an endogenous marker of ELF recovered by BAL. Concentration of ceftolozane and tazobactam in ELF will be estimated from the concentration of drug in BAL fluid, the volume of BAL fluid collected, and the ratio of urea concentration in BAL fluid to that in plasma. The following formulas represent these calculations:

Ceftolozane or tazobactam concentration in ELF = [ceftolozane or tazobactam concentration in BAL] · V_{BAL}/V_{ELF}

V_{BAL} is the volume of aspirated BAL fluid (total); V_{ELF} is $V_{BAL} \cdot [urea]_{BAL}/[urea]_{plasma}$, where $[urea]_{BAL}$ is the concentration of urea in the BAL fluid (supernatant) and $[urea]_{plasma}$ is the concentration of urea in the plasma specimens.

BAL and blood samples collected at the time of BAL sampling will be evaluated for both CRP and albumin to identify any potential association between patients' inflammatory response and ceftolozane/tazobactam penetration into the ELF. Cytology counts will be conducted on BAL samples.

11. ASSESSMENT OF SAFETY

Safety will be assessed through collection of AEs, laboratory evaluations (hematology, chemistry and coagulation), vital signs, and physical examinations.

Adverse events and SAEs will be collected from administration of the first dose of study medication through the last study contact.

11.1. Definition of Adverse Events

11.1.1. Adverse Events

An AE is any untoward medical occurrence in a patient or clinical trial subject administered a pharmaceutical product that does not necessarily have to have a causal relationship with this treatment. An AE can therefore be any unfavorable and unintended sign (including an abnormal laboratory finding), symptom, or disease temporally associated with the use of a medicinal product, whether or not related to the medicinal product.

- Adverse events may be new events or may be pre-existing conditions that have become aggravated or have worsened in severity or frequency
- Adverse events may be clinically significant changes from baseline in laboratory tests or other diagnostic investigations, and abnormal physical examination findings.

Pregnancy is not an AE; however, if a patient or female partner of a male patient becomes pregnant during the conduct of the study, the Investigator must notify Cubist according to the procedures in Section 11.5.2.

11.1.2. Serious Adverse Event

A SAE is any adverse experience occurring at any dose that results in any of the following outcomes:

- Death;
- Life-threatening experience
  - Note: "Life-threatening" refers to a situation in which the patient was at risk of death *at the time of the event*; it does not refer to an event which might have caused death if it were more severe;
- Requires inpatient hospitalization or prolongation of existing hospitalization
  - Note: Adverse events requiring hospital admissions that are less than 24 hours in duration do not meet this criterion. A scheduled hospitalization for a pre-existing condition that has not worsened during participation in the study does not meet this criterion. Pre-planned hospitalizations for an elective medical/surgical procedure or routine check-ups do not meet this criterion;
- Results in persistent or significant disability or incapacity;
- Is a congenital anomaly or birth defect;

- Is considered to be an important medical event.
  - Note: Important medical events are those that may not be immediately life-threatening or result in death or hospitalization but may jeopardize the patient or may require intervention to prevent one of the outcomes listed in the definition above.

11.1.3. Overdose

For this study, an overdose of ceftolozane/tazobactam is considered to be any dose that is higher than the protocol-specified dose for the patient's age cohort. Any overdose must be reported to Cubist as described in Section 11.5.

11.2. Monitoring of Adverse Events

Each patient will be monitored for the occurrence of AEs, including SAEs, beginning immediately after administration of the first dose of study medication. Each patient will be followed for safety monitoring until the follow-up visit in the trial as described in the schedule of assessments (see Table 2).

Patients will be questioned and/or examined by the Investigator or a qualified designee for evidence of AEs. The questioning of patients with regard to the possible occurrence of adverse events will be generalized such as, "How have you been feeling since your last visit?" The presence or absence of specific AEs should not be elicited from patients.

Patients having AEs will be monitored with relevant clinical assessments and laboratory tests, as determined by the Investigator.

Adverse events, actions taken as a result of AEs, and follow-up results must be recorded in the eCRF, as well as in the patient's source documentation. Follow-up laboratory results should be filed with the patient's source documentation.

For all SAEs and AEs that require the patient to be discontinued from the trial, relevant clinical assessments and laboratory tests will be repeated as clinically appropriate, until final resolution or stabilization of the event(s).

11.3. Monitoring of Laboratory Assessments

All laboratory assessments will be performed at a local laboratory. The clinical laboratory values will be reported to the investigator who will review them for significance and consideration as an AE.

11.4. Assessment of Adverse Events

11.4.1. Assessment of Severity

The term "severe" is often used to describe the intensity (severity) of a specific event (as in mild, moderate, or severe myocardial infarction); the event itself, however, may be of relatively minor medical significance (such as severe nausea). This is not the same as "serious," which is based on patient/event outcome or action criteria usually associated with events that pose a threat to a patient's life or functioning. The severity of AEs will be assessed according to the following definitions:

- **Mild**: the AE is noticeable to the patient and/or the Investigator, but does not interfere with routine activity.
- **Moderate**: the AE interferes with routine activity, but responds to symptomatic therapy or rest.
- **Severe**: the AE significantly limits the patient's ability to perform routine activities despite symptomatic therapy.

11.4.2. Assessment of Causality

A medically-qualified Investigator must assess the relationship of any AE (including SAEs) to the use of a study drug, as related or not related, based on clinical judgment and using all available information, and may include consideration of the following factors:

- Possible alternative causes of the AE, including the disease under treatment, pre-existing conditions, concomitant use of other drugs, and presence of environmental or genetic factors.
- The temporal association between drug exposure and onset of the AE.
- Whether the manifestations of the AE are consistent with known actions or toxicity of the investigational product.
- **Dechallenge**: the AE resolved or improved with stopping use of the investigational product. Judgment should be used if multiple products are discontinued at the same time.
- **Rechallenge**: the AE recurred or worsened upon re-exposure to the investigational product.

The causal relationship between a study drug and the AE will be assessed using one of the following categories:

Not Related: An AE is not associated with study drug if:

- Temporal relationship is lacking (e.g., the event did not occur within a reasonable time frame following administration of the study medication); **or**
- Other causative factors more likely explain the event (e.g., a pre-existing condition, other concomitant treatments); **or**
- Dechallenge was either not clinically indicated or did not result in clinical improvement; or
- AE did not reoccur upon rechallenge (if applicable).

Related: An AE is attributed to the study drug if:

- There is a positive temporal relationship (e.g., the event occurred within a reasonable time frame following administration of study medication); **and**
- The AE is more likely explained by the investigational product than by another cause (i.e., the AE shows a pattern consistent with current knowledge of the investigational product or the class of the investigational product), or The event resolved on dechallenge
- The event re-occurred upon rechallenge (if applicable).

11.4.3. Reference Safety Information for the Assessment of Expectedness of AEs

The Reference Safety Information (RSI) for assessing the expectedness of an adverse event for ceftolozane/tazobactam in this trial can be found in the Developmental Core Safety Information (DCSI) section of the most recent Investigator's Brochure for ceftolozane/tazobactam.

11.5. Reporting Safety Observations by the Investigator to the Sponsor

11.5.1. Reporting of Nonserious AEs

All AEs, regardless of seriousness, severity, or causal relationship to study medication, will be recorded on the AE page of the eCRF.

11.5.2. Reporting of Drug Exposure During Pregnancy

If the patient or the female partner of a male patient becomes pregnant after receiving study medication during the course of study, the Investigator must report this to the Sponsor using the Pregnancy Reporting Form within 24 hours of becoming aware of the event.

If the female partner of a male patient becomes pregnant, the Investigator must attempt to obtain consent to collect pregnancy information (including the status of the newborn, if applicable) before reporting information to the Sponsor.

If not all information on the Pregnancy Reporting Form is available at the time of the initial report, follow-up Pregnancy Reporting Forms will be completed and submitted within 24 hours of becoming aware of new information. The Investigator is required to attempt follow-up on the pregnancy until the completion of the pregnancy. The outcome of the pregnancy and the status of the newborn (if applicable) will be reported on the Pregnancy Reporting Form within 24 hours of the Investigator becoming aware.

11.5.3. Reporting of Expedited Safety Observations by the Investigator

Any occurrence of the following events or outcomes in a patient in the trial must be reported expeditiously by the Investigator or qualified designee to the Sponsor:

- SAE;
- Death of a patient;
- Overdose;
- New onset of cancer

The investigator is to report any Expedited Safety Observations from the list above to the Sponsor using the **Expedited Safety Observation Reporting Form** within **24 hours** of becoming aware of the event.

MERCK CLINICAL SAFETY

Email: PPD

Fax: PPD

Any observation reported to the Sponsor via the **Expedited Safety Observation Reporting Form** that is also an AE, is to be recorded in the eCRF, as well as in the patient's source documentation along with any actions taken. If not all information on the Expedited Safety Observation Reporting Form is available at the time of the initial report, follow-up SAE reports will be completed and submitted.

The Investigator is required to follow SAEs until resolution regardless of whether the patients are still participating in the study. Resolution is defined as:

- Resolved with or without residual effects (sequelae);
- A return to baseline for a pre-existing condition;
- The Investigator does not expect any further improvement or worsening of the event;
- Fatal outcome: If an autopsy is performed on a deceased patient, the autopsy report must be provided to the sponsor as soon as it is available.

11.6. Expedited Reporting by the Sponsor to the FDA

The Sponsor will monitor the data for safety. All SAEs that are considered unexpected and related to the study agent (SUSAR) will be reported by Cubist or designee as expedited (i.e., 7/15-Day) reports to the US FDA and to all participating investigators. In addition, Cubist or designee follows all applicable local and national regulatory requirements regarding safety reporting. Each investigator must also comply with the applicable regulatory requirements related to the reporting of SAEs to the IRB responsible for reviewing the study at their site, as well as the regulatory authority(ies) (if applicable).

11.7. Safety Notifications by the Sponsor to the Investigator

Investigators will receive prompt notification of any adverse experience associated with the use of the study medication that is both serious and unexpected, or any finding that suggests a significant risk for patients. The Investigator will promptly inform the IRB of the notification and insert the notification in the Investigator's Regulatory Binder in accordance with local regulations.

12. STATISTICAL CONSIDERATIONS

12.1. Data Collection, Processing and Reporting

The site will be supplied with the following data collection tool: a web browser address for an Electronic Data Capture (EDC) system database that has been fully validated and conforms to 21 Code of Federal Regulations (CFR) Part 11 and the Guidance for Industry on Computerized Systems Used in Clinical Trials requirements. The EDC system database will be maintained by a Contract Research Organization (CRO).

The trained Investigator site staff will enter the data required by the protocol into the eCRFs from source documents (e.g., medical records and study-specific data capture forms as needed) into the EDC system. All information on the eCRFs must be traceable to these source documents. Data recorded directly on the eCRFs will be defined before study start.

eCRFs will be completed for all patients. Informed consent, demography, inclusion/exclusion, End of Treatment and End of Study Participation eCRF pages are needed for patients who are enrolled but not treated. A clinical monitor will review the eCRFs entered by investigational staff for completeness and accuracy.

Automatic validation programs or manual checks for data discrepancies in the eCRFs may result in electronic queries generated for resolution by the investigational site. Designated investigator site staff are required to respond to these queries and make any necessary changes to the data.

All treatment-emergent AEs (events occurring from the first dose of study medication through the last study evaluation) will be recorded. Medical and surgical history and AEs will be coded using the Medical Dictionary for Regulatory Activities (MedDRA). Concomitant medications will be coded using the World Health Organization (WHO) Drug Dictionary.

Audits for quality assurance of the database may be performed according to relevant Standard Operating Procedures within the CRO or at the request of Cubist’s Quality Assurance department.

12.2. Populations for Analysis

12.2.1. PK Population

Group1: The PK population to evaluate ELF penetration will consist of patients who receive at least 4 doses (6 doses for patients with severe renal impairment [CL_{CR} 15 – 29 mL/min] at baseline) of ceftolozane/tazobactam and who provide at least one serial blood sample and one BAL sample post-completion of infusion.

Group 2: The PK population will consist of patients who receive one dose of ceftolozane/tazobactam and who provide at least three serial blood samples post-completion of infusion, inclusive of C_{max}, to be able to estimate AUC.

12.2.2. Safety Population

The Safety population will include patients who receive study medication (including partial doses).

12.2.3. Endpoints

12.2.3.1. Primary Endpoint

The primary endpoint is the characterization of plasma PK (Group 1 and Group 2), and intrapulmonary penetration that includes determination of the ELF to plasma ratio of ceftolozane and tazobactam concentrations (Group 1). Patients in Group 1 who undergo an additional BAL (at any time point post-dose through follow-up assessment) as part of standard of care may be collected and analyzed.

12.2.3.2. Secondary Endpoint

The secondary endpoint is evaluation of the safety and tolerability of ceftolozane/tazobactam in mechanically ventilated patients concurrently receiving standard antibiotic therapy for proven or suspected pneumonia. Safety will be determined by clinical review and interpretation of all adverse events and laboratory findings.

12.2.4. Interim Analysis

In Group 1, an interim population PK analysis will be conducted after 3 BAL samples at each scheduled sampling time point are available. The interim analysis will determine whether enrollment in Group 1 may stop early based on the following criteria:

- the inter-individual variability of the plasma-to-ELF penetration ratio, defined as the ratio of the clearance from plasma to ELF over the clearance from ELF to plasma in a pre-defined 3-compartment PK model, is not higher than that observed in the previous ELF study in healthy volunteers (42%CV), OR
- the Bayesian estimated minimum penetration ratio from the 15 patients is not lower than that observed in the previous ELF study in healthy volunteers (0.26).

Enrollment in Group 1 may continue beyond 25 patients if the penetration ratio (ratio and its variability) cannot be reliably estimated, e.g., the relative standard error is higher than 50%, which means that the lower boundary of the 95%CI of the parameter becomes negative and therefore meaningless. Whether to enroll beyond 25 patients and how many additional patients need to be enrolled will be determined by further simulations, and if needed, the protocol will be amended accordingly.

13. PHARMACOKINETIC ANALYSIS PLAN

A Pharmacokinetic Analysis Plan with further details will be prepared, and will be finalized before database lock and analysis of the data..

13.1.1. Pharmacokinetic Parameters

Ceftolozane/tazobactam pharmacokinetic parameters will be determined by non-compartmental pharmacokinetic analysis as allowed by the data. The plasma and ELF PK parameters may include but are not limited to:

- C_{max} – Maximum observed plasma concentration over the entire sampling interval
- T_{max} – Time of C_{max}
- C_{last} – Last quantifiable plasma concentration
- T_{last} – Time of C_{last}
- $AUC_{0\text{-}last}$ – Area under the concentration time curve from the time of the dose to the T_{last}
- AUC_{∞} – Area under the concentration versus time curve from zero to infinity
- t½ – Half-life
- V_{ss} – Volume of distribution at steady state (plasma only)
- CL – Plasma clearance (plasma only)
- $AUC_{0\text{-}tELF}$: mean $AUC_{0\text{-}tPlasma}$ – Percent penetration into ELF
- C_{tELF}: $C_{tPlasma}$ – ELF-to -plasma concentration ratio at each time point

Since one BAL sample per patient will be collected, non-compartmental analysis for ELF PK will be based on the geometric mean of ELF concentration versus time. If any other summary statistics such as mean or median ELF concentrations is considered appropriate based on the data, it may be used with the justification provided.
In addition, PK data obtained from all groups will be pooled for a population PK analysis, as allowed by the data. These results will be reported separately.

13.1.2. Below Limit of Quantification Values/Missing Values

All pre-dose below limit of quantification (BLQ) values in period 1 will be set to zero. Missing or BLQ values obtained after the first quantifiable concentration will be replaced by a period.
Sampling Times
Actual blood draw times will be used to calculate PK parameters. For the final analysis, tables for the PK calculations will be based on actual times.

13.1.3. Pharmacokinetic/Pharmacodynamic Analysis (Group 1)

BAL and blood samples collected at the time of BAL sampling will also be tested for inflammatory markers (CRP and albumin) to evaluate the potential correlation between these inflammatory markers and ELF penetration. Cytology counts will also be performed on BAL samples.

13.2. Presentation of Data

Data will be presented in tables and listings. Listings may include but are not limited to: patient ID, dose administered, body weight, drug concentrations, time points and individual derived PK parameters. Summary tables may include but are not limited to the number of patients, arithmetic means, standard deviations (SD), coefficient of variation expressed as percentage (CV%), minimum, median and maximum values, and geometric mean for individual parameters.

13.3. PK Data Reporting

The clinical study report will provide the following information:

- Deviations from described PK analysis, if any
- Results and discussion of the pharmacokinetic parameters
- Study conclusions

13.4. Data Displays

Data displays may include but are not limited to:

<u>In-text tables</u>

- Summary statistics (n, mean, CV%, SD, median, minimum, maximum, geometric mean, and geometric SD) of plasma PK parameters for each analyte
- ELF PK parameters from the geometric mean ELF concentration-time profile for each analyte
- ELF to plasma AUC ratio for each analyte
- Summary statistics (n, mean, CV%, SD, median, minimum, maximum, geometric mean, and geometric SD) of the ratio of ELF/Plasma concentration data for each analyte
- Summary statistics (n, mean, CV%, SD, median, minimum, maximum, geometric mean, and geometric SD) of plasma and ELF concentration-time data for each analyte

<u>In-text graphs</u>

- Geometric mean plasma concentration-time profile and ELF concentrations-time profiles (with geometric SD) for each analyte

<u>Appendix</u>

- Mean plasma concentration-time profile and mean of ELF concentrations-time profiles (with SD) for each analyte
- Individual plasma concentration-time profiles (linear-linear and log-linear)
- Listing of individual concentration-time data and pharmacokinetic parameters by patient and analyte
- Exploratory plots such as ELF levels of analytes versus inflammatory markers (ELF levels of CRP and albumin)
- Summary statistics (n, mean, CV%, SD, median, minimum, maximum, geometric mean, and geometric SD) of ELF and corresponding plasma concentrations of CRP and albumin, and ELF cytology at each time point.

14. STATISTICAL ANALYSIS PLAN

A Statistical Analysis Plan with further details will be prepared, and will be finalized before database lock and analysis of the data.

14.1. General Methodology

The safety and tolerability of IV ceftolozane/tazobactam in Group 1 and Group 2 patients will be evaluated in separate Safety populations. Descriptive statistics for continuous variables will include number of patients, mean, SD, median, minimum and maximum. Categorical variables will include number and percent of the patients in each category. Adverse event data will be tabulated by Group (1 or 2) and dose (if not all patients received the same dose). Clinical laboratory data will be presented at baseline and at follow-up. Vital signs data will be presented at baseline, each study day, and at follow-up.

Descriptive statistics will be used to guide decisions as to the clinical relevance of findings. No formal hypothesis tests are planned. Table and listing shells will be provided in a separate document.

14.1.1. Baseline Definitions

For all analyses, baseline will be defined as the most recent measurement prior to the first administration of study drug, unless otherwise specified.

14.1.2. Multiple Comparisons/Multiplicity

Multiplicity is not of concern for this study with a descriptive interpretation of PK and safety endpoints. No formal statistical hypotheses testing will be performed.

14.1.3. Computing Environment

All statistical analyses will be performed using SAS statistical software Version 9.1.3., unless otherwise noted. Adverse events will be coded using the MedDRA. Concomitant medications will be coded using the WHO Drug Dictionary.

14.1.4. Patient Disposition

Patient disposition data will be summarized for all patients by Group (1 or 2). Number and percent of patients enrolled, patients who completed the study, patients who discontinued treatment, and patients who discontinued from the study will be tabulated together with the reasons for premature discontinuation of study medication and discontinuation from study participation. A listing of all patients, along with data on their disposition, will be provided.

14.1.5. Demographics and Baseline Data

Demographic information and baseline clinical data will be summarized by Group (1 or 2). Demographic and baseline characteristic variables will be presented in listings and in summary tables. Medical history, medication history, and screening laboratory tests will be presented in listings only.

In all patients who undergo an 8-hour urine collection, an exploratory analysis will be performed to compare baseline measured CL_{CR} with baseline estimated CL_{CR} (as calculated by the Cockcroft-Gault equation) and will be summarized by descriptive statistics.

Baseline will be defined as the most recent measurement prior to the administration of study drug, unless otherwise specified.

14.1.6. Study Drug Exposure

Dosing information for each patient will be summarized as descriptive statistics for the total amount of study drug received by Group (1or 2). A data listing of the dosing information for each patient, including dose interruptions, will also be provided.

14.1.7. Secondary Endpoint Analysis

The safety and tolerability of IV ceftolozane/tazobactam in critically ill patients will be evaluated in the Safety population. The safety evaluation will be based on clinical review of the following safety parameters:

- Incidence of AEs and SAEs
- AEs and SAEs by relationship to study drug
- AEs and SAEs by severity
- Deaths
- Premature discontinuation from the study due to an AE, regardless of relationship to study medication;
- Clinical laboratory data;
- Vital signs;
- Concomitant medications

14.1.8. Adverse Events

All AEs will be coded using MedDRA and displayed in tables and data listings by system organ class (SOC) and preferred term (PT).

Analyses of AEs will be performed for those events that are considered treatment-emergent, where treatment-emergent is defined per protocol as any AE with onset after the administration of study medication through the end of the study, or any event that was present at baseline but worsened in intensity or frequency. In this study, only treatment-emergent AEs are captured in the database.

The number and percentage of patients with the following treatment-emergent adverse event categories will be summarized by Group (1or 2): at least 1 adverse event, AEs by maximum severity, AEs by greatest relationship to study drug, at least 1 treatment-related severe AE, at least 1 SAE, at least 1 treatment related SAE, at least 1 AE leading to premature treatment discontinuation, at least 1 treatment-related AE leading to premature treatment discontinuation,

at least 1 AE leading to early study withdrawal, at least 1 treatment-related AE leading to early study withdrawal, AE leading to death and treatment-related AE leading to death. In addition, summary tables will be provided for all AEs by SOC and PT, AEs by severity and AEs by relationship to study drug. For all summary tables of AE incidences, SOC and PTs will be presented in alphabetical order.

All AEs and SAEs will be tabulated.. Laboratory data will be summarized by type of laboratory test. Descriptive statistics of laboratory values, vital signs and changes from baseline will be summarized. Abnormalities detected during the physical examination will be summarized as AEs in frequency tables.
In the event of missing data, a conservative approach will be taken. For example, if the relationship of an AE is not recorded in the eCRF the event will be considered to be treatment related, or if the severity of the event is missing the event will be considered severe.

No formal hypotheses testing of AE incidence rates will be performed.

All AEs occurring on-study will be listed in patient data listings.

By-patient AE listings will be provided for the following subsets, as applicable: patient deaths; serious adverse events; and adverse events leading to premature study drug discontinuation.

14.1.9. Laboratory Data

Clinical laboratory values will be expressed in SI units.

The actual value and change from baseline at Follow-up will be summarized for each clinical laboratory parameter, including hematology, clinical chemistry and coagulation. In the event of repeat values, the last non-missing value per visit will be used.

All laboratory data will be provided in data listings. Laboratory results outside the normal ranges will be flagged in these listings.

14.1.10. Vital Signs

The actual value and change from baseline to each on-study evaluation will be summarized for vital signs. Vital sign measurements will be presented for each patient in a data listing.

14.1.11. Concomitant Medications

Concomitant medications will be coded using the WHO Drug Dictionary. The use of concomitant medications will be included in a by-patient data listing. Tabular summaries will be presented.

14.1.12. Sample Size and Power Considerations

Group 1: Approximately 25 mechanically ventilated patients concurrently receiving standard antibiotic therapy for proven or suspected pneumonia. Each patient will undergo one BAL procedure with 5 patients assigned to each of the 5 sampling time points.

Group 2: 8 - 10 critically ill patients with $CL_{CR} \geq 180$ mL/min.

The sample size was chosen based primarily on empirical considerations and feasibility, and is considered sufficient to meet the study objectives. This study is an exploratory PK study and is not powered to perform inferential statistics.

With 25 subjects in Group 1, the probability of detecting at least one event with a true event of 5% is 72%. With 10 subjects in Group 2, the probability of detecting at least one event with a true event of 5% is 40% [11].

14.1.13. Evaluation Criteria

Safety will be assessed through monitoring adverse events from the start time of study drug administration to the end of study. Any serious adverse events that occur during the study will be reported and recorded.

15. DIRECT ACCESS TO SOURCE DATA/DOCUMENTS

15.1. Study Monitoring

Before an investigational site can enter a patient into the study, a representative of Cubist will assess the investigational study site to:

- Determine the adequacy of the facilities
- Discuss with the investigator(s) and other personnel their responsibilities with regard to protocol adherence, and the responsibilities of Cubist or its representatives. This will be documented in a Clinical Study Agreement between Cubist and the investigator.

During the study, a monitor from Cubist or representative will have regular contacts with the investigational site, for the following:

- Provide information and support to the investigator(s)
- Confirm that facilities remain acceptable
- Confirm that the investigational team is adhering to the protocol, that data are being accurately recorded in the case report forms, and that investigational product accountability checks are being performed
- Perform source data verification. This includes a comparison of the data in the case report forms with the patient's medical records at the hospital or practice, and other records relevant to the study. This will require direct access to all original records for each patient (e.g. clinic charts).
- Record and report any protocol deviations not previously sent to Cubist.
- Confirm AEs and SAEs have been properly documented on CRFs and confirm any SAEs have been forwarded to Cubist and those SAEs that met criteria for reporting have been forwarded to the IRB.

The monitor will be available between visits if the investigator(s) or other staff needs information or advice.

15.1.1. Protocol Deviations/Amendments

Any deviation from the protocol that has not been approved by Cubist and the IRB/EC could result in a discontinuation from the study of the center involved. Both Cubist and the IRB/EC that granted the original approval of the study prior to their implementation (unless only logistical or administrative aspects of the trial are involved) must approve any amendment(s) to the protocol.

However, in the event of any medical emergency, the Investigator is free to institute any medical procedure s/he deems appropriate for proper management of the patient. Such events must be promptly reported to Cubist and recorded in the source documents.

15.1.2. Discontinuation of the Study

Cubist may stop the study at any time on the basis of new information regarding safety or efficacy. Additionally, Cubist may terminate the study if progress is unsatisfactory.

15.2. Audits and Inspections

Authorized representatives of Cubist, a regulatory authority, or an IRB/EC may visit the site to perform audits or inspections, including source data verification. The purpose of a Cubist audit or inspection is to systematically and independently examine all study-related activities and documents to determine whether these activities were conducted, and data were recorded, analyzed, and accurately reported according to the protocol, Good Clinical Practice guidelines of the International Conference on Harmonization, and any applicable regulatory requirements. The Investigator should contact Cubist immediately if contacted by a regulatory agency about an inspection.

Audits for quality assurance of the database may be performed according to relevant Standard Operating Procedures at the request of Cubist’s Quality Assurance department.

15.3. Institutional Review Board (IRB)

The Principal Investigator must obtain IRB approval for the investigation. Initial IRB approval, and all materials approved by the IRB for this study including the study protocol and any amendments, the Informed Consent Form and any other written documents to be provided to the patient, and recruitment materials must be maintained by the Investigator and made available for inspection.

16. QUALITY CONTROL AND QUALITY ASSURANCE

To ensure compliance with Good Clinical Practices and all applicable regulatory requirements, Cubist may conduct a quality assurance audit.

17. ETHICS

17.1. Ethics Review

The final study protocol, including the final version of the Informed Consent Form, must be approved or given a favorable opinion in writing by an IRB/EC as appropriate. The investigator must submit written approval to Cubist before he or she can enroll any patient/patient into the study.

The Investigator is responsible for informing the IRB/EC of any amendment to the protocol in accordance with local requirements. In addition, the IRB/EC must approve all advertising used to recruit patients for the study. The protocol must be re-approved by the IRB/EC upon receipt of amendments and annually, in accordance with local regulations.

The Investigator is also responsible for providing the IRB/EC with reports of any reportable serious adverse drug reactions from any other study conducted with the investigational product. Cubist will provide this information to the Investigator.

Progress reports and notifications of serious adverse drug reactions will be provided to the IRB/EC according to local regulations and guidelines.

17.2. Ethical Conduct of the Study

The study will be performed in accordance with the protocol, ethical principles that have their origin in the Declaration of Helsinki, and are consistent with International Conference on Harmonization (ICH) E6 Good Clinical Practice (GCP): Consolidated Guideline, 21 CFR 312.120, and any applicable local regulations.

17.3. Written Informed Consent

The Principal Investigator(s) at each center will ensure that the patient (or the legal representative when legally acceptable by local country and institution specific guidelines) is given full and adequate oral and written information about the nature, purpose, possible risk and benefit of the study. Patients must also be notified that they are free to discontinue from the study at any time. The patient should be given the opportunity to ask questions and allowed time to consider the information provided.

The patient’s (or legal representative’s) signed and dated informed consent must be obtained before conducting any study procedures.

The Principal Investigator(s) must maintain the original, signed Informed Consent Form. A copy of the signed Informed Consent Form must be given to the patient or legal representative and the original retained in the source documents of the study participant.

18. DATA HANDLING AND RECORDKEEPING

18.1. Inspection of Records

Cubist will be allowed to conduct site visits to the investigation facilities for the purpose of monitoring any aspect of the study. The Investigator agrees to allow the monitor to inspect the drug storage area, study drug stocks, drug accountability records, patient charts and study source documents, and other records relative to study conduct.

18.2. Retention of Records

The Principal Investigator must maintain all documentation relating to the study for a period of 2 years after the last marketing application approval, or if not approved 2 years following the discontinuance of the test article for investigation. If it becomes necessary for Cubist or health authorities to review any documentation relating to the study, the Investigator must permit access to such records.

19. PUBLICATION POLICY

Cubist may publish the results of company-sponsored studies in a timely manner, regardless of whether the outcomes are perceived as positive, neutral, or negative. Publications will adhere to external guidelines, including the guidelines adopted by the International Society for Medical Publication Professionals and the International Committee of Medical Journal Editors, subject to taking appropriate steps to protect Cubist's intellectual property rights. All publications will comply with Cubist policies, including those described in MA-SBP-126 (Scientific, Technical, and Medical Publications) and MA-SBP-113 (Development and Review of Publications).

20. LIST OF REFERENCES

1. Sievert DM, Ricks P, Edwards JR, et al. Antimicrobial-resistant pathogens associated with healthcare-associated infections: summary of data reported to the National Healthcare Safety Network at the Centers for Disease Control and Prevention, 2009-2010. *Infect Control Hosp Epidemiol*. 2013;34:1-14.
2. Jones, RN. Microbial etiologies of hospital-acquired bacterial pneumonia and ventilator associated bacterial pneumonia. *Clin Infect Dis* 2010; 51:S81-7.
3. Ambrose PG, Bhavnani SM, Ellis-Grosse EJ, Drusano GL. Pharmacokinetic-pharmacodynamic considerations in the design of hospital-acquired or ventilator-associated bacterial pneumonia studies: look before you leap! *Clin Infect Dis*. 2010;51 Suppl 1:S103-10.
4. Chandorkar G, Huntington JA, Gotfried MH, Rodvold KA, Umeh O. Intrapulmonary penetration of ceftolozane/tazobactam and piperacillin/tazobactam in healthy adult subjects. *J Antimicrob Chemother*. 2012;67(10):2463-9.
5. Lamer C, de Beco V, Soler P, et al. Analysis of vancomycin entry into pulmonary lining fluid by bronchoalveolar lavage in critically ill patients. *Antimicrob Agents Chemother*. 1993;37(2):281-286.
6. Povoa P, Coelho L, Almeida E, et al. C-reactive protein as a marker of infection in critically ill patients. *Clin Microbiol Infect*. 2005;11(2):101-8.
7. Claus BOM, Hoste EA, Colpaert K, Robay H, Decruyenaere J, DeWaele JJ. Augmented renal clearance is common finding with worse clinical outcome in critically ill patients receiving antimicrobial therapy. *J Crit Care*. 2013;28(5)695-700.
8. Noel GJ, Strauss R, Shah A, et al. Ceftobiprole (BPR) versus ceftazidime (CAZ) combined with linezolid (LZD) for treatment of patients with nosocomial pneumonia (NP). Abstracts of the 48th Interscience Conference on Antimicrobial Agents and Chemotherapy and the Infectious Diseases Society of America 46th Annual Meeting, Washington, DC, 2008. Washington, DC, USA: American Society for Microbiology; Abstract K-486, p. 444.
9. Fuster-Lluch O, Geronimo-Pardo M, Peyro-Garcia R, Lizan-Garcia M. Glomerular hyperfiltration and albuminuria in critically ill patients. *Anaesth Intensive Care*. 2008;36:674-680.
10. Claus B, Colpaert K, Hoste E, Decruyenaere J, DeWaele J. Increased glomerular filtration in the critically ill patient receiving anti-infective treatment. *Crit Care*. 2010; 14(Suppl 1):P509.
11. Schoenfeld D. Statistical considerations for pilot studies. Int. J. Radiation Oncology Biol. Phys. Vol 6: 371-374, 1980.
12. Knaus WA, Draper EA, Wagner DP, Zimmerman JE. APACHE II: a severity of disease classification system. *Crit Care Med*. 1985;13(10):818-29.

.

21. APPENDICES

21.1. Appendix A: Acute Physiology and Chronic Health Evaluation (APACHE) II Severity of Disease Classification System

The APACHE II Score should be calculated during screening using the worst patient values obtained in the 24 hours prior to enrollment. The total APACHE II Score is the sum of (A) the Total Acute Physiology Score, (B) Age Points, and (C) Chronic Health Points [12].

A. Total Acute Physiology Score: Sum of the 12 individual variable points as follows:

Physiologic Variable	High Abnormal Range					Low Abnormal Range			
	**+4**	**+3**	**+2**	**+1**	**0**	**+1**	**+2**	**+3**	**+4**
Temperature - rectal (°C)	≥4	39 - 40.9		38.5 -38.9	36 - 38.4	34 - 35.9	32 - 33.9	30 - 31.9	≤29.9
Mean Arterial Pressure (mmHg)	≥160	130 - 159	110 - 129		70 - 109		50 - 69		≤49
Heart Rate (ventricular response)	≥180	140 - 179	110 - 139		70 - 109		55 - 69	40 - 54	≤39
Respiratory Rate<br>(non-ventilated or ventilated)	≥50	35 - 49		25 - 34	12 - 24	10 - 11	6 - 9		≤5
Oxygenation: A-aDO_2 **or** PaO_2 (mmHg)<br>a. FIO_2 ≥0.5 record A-aDO_2<br>b. FIO_2 <0.5 record PaO_2	≥500	350 - 499	200 - 349		<200<br>PO_2>70	PO_2 61 - 70		PO_2 55 - 60	PO_2<55
Arterial pH (preferred)<br>Serum HCO_3 (venous mEq/L)<br>(not preferred, use if no ABGs)	≥7.7<br>≥52	7.6 - 7.69<br>41 - 51.9		7.5 - 7.59<br>32 - 40.9	7.33 - 7.49<br>22 - 31.9		7.25 - 7.32<br>18 - 21.9	7.15 - 7.24<br>15 - 17.9	<7.15<br><15
Serum Sodium (mEq/L)	≥180	160 - 179	155 - 159	150 - 154	130 - 149		120 - 129	111 - 119	≤110
Serum Potassium (mEq/L)	≥7	6 - 6.9		5.5 - 5.9	3.5 - 5.4	3 - 3.4	2.5 - 2.9		<2.5
Serum Creatinine (mg/dL)<br>(x 2 for acute renal failure)	≥3.5	2 - 3.4	1.5 - 1.9		0.6 - 1.4		<0.6		
Hematocrit (%)	≥60		50 - 59.9	46 - 49.9	30 - 45.9		20 - 29.9		<20
White Blood Count (total[in 1000s]/mm^3)	≥40		20 - 39.9	15 - 19.9	3 - 14.9		1 - 2.9		<1
Glasgow Coma Score (GCS)<br>(Score = 15 minus actual GCS)									

B. Age Points: Assign points to age as follows:

- 0 points for ≤44 years
- 2 points for 45 - 54 years
- 3 points for 55 - 64 years
- 5 points for 65 - 74 years
- 6 points for ≥75 years

C. Chronic Health Points: If the patient has a history of severe organ system insufficiency or is immunocompromised as defined below, assign points as follows:

- 5 points for non-operative or emergency postoperative patients
- 2 points for elective postoperative patients
- ***Definitions*****:** organ insufficiency or immunocompromised state must have been evident **prior** to this hospital admission and conform to the following criteria:
    - **Liver –** biopsy proven cirrhosis and documented portal hypertension; episodes of past upper GI bleeding attributed to portal hypertension; or prior episodes of hepatic failure/encephalopathy/coma.
    - **Cardiovascular** – New York Heart Association Class IV.
    - **Respiratory** – Chronic restrictive, obstructive, or vascular disease resulting in severe exercise restriction (i.e., unable to climb stairs or perform household duties; or documented chronic hypoxia, hypercapnia, secondary polycythemia, severe pulmonary hypertension (>40 mmHg), or respirator dependency.
    - **Renal** – receiving chronic dialysis.
    - **Immunocompromised –** the patient has received therapy that suppresses resistance to infection (e.g., immunosuppression, chemotherapy, radiation, long term or recent high dose steroids, or has a disease that is sufficiently advanced to suppress resistance to infection, e.g., leukemia, lymphoma, AIDS).
